# Replacing Antibiotics with Synergistic Probiotics–Microalgae Consortium in Mud Crab (*Scylla paramamosain*) Larviculture: Transcriptomic Evidence for Enhanced Innate Immunity, Oxidative Stress Response, and Metabolic Adaptability

**DOI:** 10.3390/antibiotics15050498

**Published:** 2026-05-16

**Authors:** Xiaokang Lv, Lingbo Ma, Bo Liu, Yongxu Cheng, Wei Wang, Baojun Tang, Cunxin Sun, Yin Fu

**Affiliations:** 1 Key Laboratory of East China Sea and Oceanic Fishery Resources Exploitation, East China Sea Fisheries Research Institute, Chinese Academy of Fishery Sciences, Ministry of Agriculture and Rural Affairs, Shanghai 200090, China; lvxk@ecsf.ac.cn (X.L.); malb@ecsf.ac.cn (L.M.); wangw@ecsf.ac.cn (W.W.); tangbj@ecsf.ac.cn (B.T.); 2 Wuxi Fisheries College, Nanjing Agricultural University, Wuxi 214081, China; liub@ffrc.cn (B.L.); suncx@ffrc.cn (C.S.); 3 Key Laboratory of Freshwater Fisheries and Germplasm Resources Utilization, Freshwater Fisheries Research Center, Chinese Academy of Fishery Sciences, Ministry of Agriculture and Rural Affairs, Wuxi 214081, China; 4 College of Fisheries and Life Sciences, Shanghai Ocean University, Shanghai 201306, China; yxcheng@shou.edu.cn

**Keywords:** *Scylla paramamosain*, larviculture, probiotics, microalgae, antibiotic alternative, transcriptomics, innate immunity, oxidative stress

## Abstract

**Background/Objectives:** Antibiotics are routinely used in crustacean larviculture to mitigate bacterial infections, yet their widespread application compromises larval ontogeny. Probiotics and microalgae offer sustainable alternatives, but their combined molecular effects in crustacean larvae remain poorly characterized. This study aimed to evaluate the physiological and transcriptomic impacts of a probiotics–microalgae consortium versus antibiotics in mud crab (*Scylla paramamosain*) zoea, with the goal of elucidating mechanisms underlying improved larval development and identifying potential antibiotic alternatives. **Methods:** *Scylla paramamosain* larvae were reared under five treatments: clear water control (CN), microalgae alone (MA), probiotics alone (PB), a probiotics–microalgae consortium (PB-MA), and the antibiotic (AB) florfenicol. Samples were collected at 6 h and 24 h post-treatment during the first (Z1) and third (Z3) zoeal stages. Growth performance was assessed via survival and larval stage index, and multi-time point transcriptomic sequencing was performed to analyze dynamic gene expression profiles. **Results:** The PB-MA consortium significantly enhanced stage-specific survival from Z3 to Z5 and accelerated developmental progression compared to control and antibiotic groups. Transcriptomic analysis revealed from 492 to 2854 differentially expressed genes across treatments. PB-MA treatment was associated with the sustained upregulation of immune-related pathways (lysosome and Toll/Imd signaling), oxidative stress responses (peroxisome and glutathione metabolism), and energy metabolism (TCA cycle and carbon metabolism), whereas antibiotics predominantly suppressed these pathways. Key candidate genes, including *NPC1*, *NAGA*, *ACOX1*, *HAO1*, *MUT*, and *PK*, were prominently induced in PB-MA-treated larvae. **Conclusions:** The probiotics–microalgae consortium enhances basal immunity, antioxidant capacity, and metabolic adaptability in mud crab larvae at the molecular level. These findings provide transcriptomic evidence supporting the replacement of antibiotics with synergistic microbial consortia in sustainable crustacean larviculture.

## 1. Introduction

Antibiotics have long been a cornerstone of disease management in aquatic larviculture, where high stocking densities and the physiological immaturity of early life stages render fish and shellfish larvae exceptionally vulnerable to bacterial infections [1]. Broad-spectrum antibiotics such as oxytetracycline [2], florfenicol [3], and enrofloxacin [4] are routinely administered either prophylactically or therapeutically to mitigate mortality from ubiquitous pathogens such as *Vibrio*, *Aeromonas*, and *Edwardsiella* [5]. While this practice reduces acute losses, widespread and indiscriminate use has become a pressing concern. A substantial fraction of administered drugs accumulates in the rearing water and biofilms, exerting continuous selective pressure that drives the emergence and dissemination of antibiotic-resistant bacteria and resistance genes within aquaculture ecosystems [6]. Beyond public health risks associated with horizontal gene transfer, emerging evidence indicates that antibiotic exposure profoundly disrupts the resident microbiota of both the rearing environment [1] and the larval host [7,8]. Such perturbations favor the proliferation of opportunistic pathogens while depleting beneficial commensals crucial for nutrient acquisition [9] and immune ontogeny [10]. Consequently, reliance on antibiotics not only threatens long-term drug efficacy but paradoxically compromises larval robustness by destabilizing microbial communities that are essential for healthy development [11]. This recognition has fueled an urgent search for sustainable hatchery strategies that decouple disease control from escalating bioinsecurity [12].

Among the most promising alternatives are probiotic microorganisms and microalgae, applied individually or as a synergistic consortium [13]. Probiotic strains, particularly lactic acid bacteria [14] and members of the *Roseobacter* clade [15], confer protection against prominent pathogens like *Vibrio anguillarum* [16], *V. harveyi* [17], and *Aeromonas hydrophila* [18] through competitive exclusion, production of antimicrobial metabolites, and immunomodulation. Supplementation also enhances innate immune parameters (e.g., phagocytic activity, complement pathway) [19,20,21,22] and accelerates gastrointestinal development, digestive enzyme expression, and intestinal epithelial barrier integrity, thereby improving nutrient assimilation during the critical first-feeding window [23].

Concurrently, microalgae are indispensable in larviculture food webs, offering nutritional [24] and immunomodulatory benefits [25] that extend far beyond their traditional role as live feed. Dietary inclusion of diatoms like *Navicula* sp. and *Chaetoceros calcitrans* provides bioavailable essential long-chain polyunsaturated fatty acids (LC-PUFAs), including EPA and DHA, which are crucial for neural development and cell membrane integrity in larvae [26]. The complex biochemical matrix of microalgae encompasses carotenoids, phycobiliproteins, and sulfated polysaccharides that function as potent antioxidants and immunostimulants [27]. Microalgal extracts themselves possess direct antimicrobial bioactivity: compounds from *Skeletonema costatum* inhibit hemolysin production and attenuate *Vibrio harveyi* virulence, reducing larval mortality without selecting for antimicrobial resistance [28]. Microalgal supplementation also upregulates immune-related genes, elevates serum immunoglobulin and lysozyme activity, and improves stress tolerance and survival [29,30,31]. Microalgae also serve as vectors for beneficial microbes: *Tetraselmis suecica* and *Nannochloropsis oculata* can be colonized by antagonistic probiotics like *Phaeobacter gallaeciensis*, creating a functional consortium that reduces *Vibrio anguillarum* loads within the feed chain [32].

The mud crab (*Scylla paramamosain*) is a highly valued economic species in Southeast Asia due to its fast growth and nutritional quality [33]; however, the larval supply often constrains industry production [34]. In crustacean larviculture, disease prevention is critical for production success. Current microalgae applications in mud crab hatcheries primarily focus on nutritional enrichment [26] rather than systematic disease control. Our previous work interpreted the dynamics of the *S. paramamosain* zoea microbiome and highlighted the potential of optimizing probiotics such as *Rhodobacterales* and *Lactobacillus* to enhance early life-stage growth [34]. Although individual uses of microalgae [28] and probiotics [35] show promise for disease control, research on their combined use as an antibiotic alternative remains very limited in crustacean larviculture. The effects of combined microalgae–probiotic treatments on larval growth, development, and disease resistance warrant further evaluation.

Over the past decade, RNA sequencing has significantly advanced investigations into environmental influences on aquatic larvae [36], proving particularly valuable for species with limited genomic resources [37] by enabling de novo transcriptome assembly and identification of immune- and growth-related genes [38,39]. Time-course transcriptomic analyses permit high-resolution tracking of gene expression dynamics at multiple intervals following exposure, offering enhanced sensitivity in delineating regulatory networks underpinning survival and stress resilience [40]. Our previous study successfully utilized multi-tissue, multi-time point transcriptomics to delineate temporally coordinated metabolic reprogramming in *S. paramamosain* during starvation [33]. Therefore, RNA-seq-based approaches are indispensable for unraveling the molecular mechanisms by which biotic interventions fortify physiological and immunological health in aquatic larviculture.

In this study, a multi-time point dynamic transcriptomic analysis was performed on *S. paramamosain* larvae reared under five conditions: a clear water control (CN), microalgae alone (MA), probiotics alone (PB), a probiotics–microalgae consortium (PB-MA), and an antibiotic group (AB) administered via water treatment. Samples were collected at four critical time points during the first (Z1) and third (Z3) zoeal stages for comprehensive sequencing. The Z1 and Z3 stages were selected because Z1 represents the critical first-feeding window when larvae are acutely sensitive to microbial cues, while Z3 marks a transitional phase characterized by accelerated organogenesis and elevated metabolic demand. Transcriptional responses at these two stages are thus likely to capture both immediate and developmentally programmed adaptive strategies. The objectives were to molecularly evaluate these sustainable antibiotic alternatives and investigate dynamic gene expression profiles underlying larval health in response to different rearing strategies. Our results systematically reveal, for the first time, the molecular mechanisms by which the probiotics–microalgae consortium enhances basal immunity, antioxidant defenses, and metabolic adaptability in crustacean larvae. Furthermore, they provide solid molecular evidence for developing effective antibiotic alternatives in aquaculture and identify synergistic pathways that serve as potential targets for next-generation functional feeds or microbial management strategies in mud crab and other commercially important marine invertebrates.

## 2. Results

### 2.1. Growth Performance

**Figure 1:**
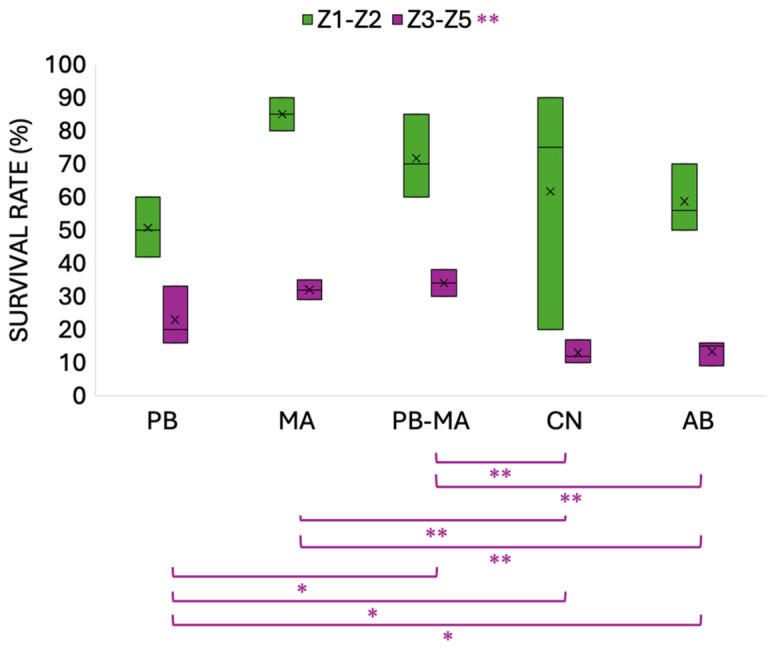
Survival of *Scylla paramamosain* zoea affected by different larviculture treatments in Z1–Z2 and Z3–Z5, respectively. PB, probiotics alone; MA, microalgae alone; PB-MA, probiotics–microalgae consortium; CN, clear water control; AB, antibiotics alone. n = 12. * *p* < 0.05; ** *p* < 0.01.

Figure 1 illustrates the changes in survival rates of *Scylla paramamosain* larvae at different developmental stages across treatment groups in the present experiment. Stage-specific survival rates revealed no significant difference in the survival rate of individuals completing metamorphosis to the Z2 stage. From the Z3 to Z5 stages, the PB-MA group exhibited the highest stage-specific survival rate, which was significantly higher than that of the control group (*p* < 0.01), the AB group (*p* < 0.01), and the PB group (*p* < 0.05). The mean survival rate of larvae in the PB-MA group completing metamorphosis to Z5 was 34%.

**Figure 2:**
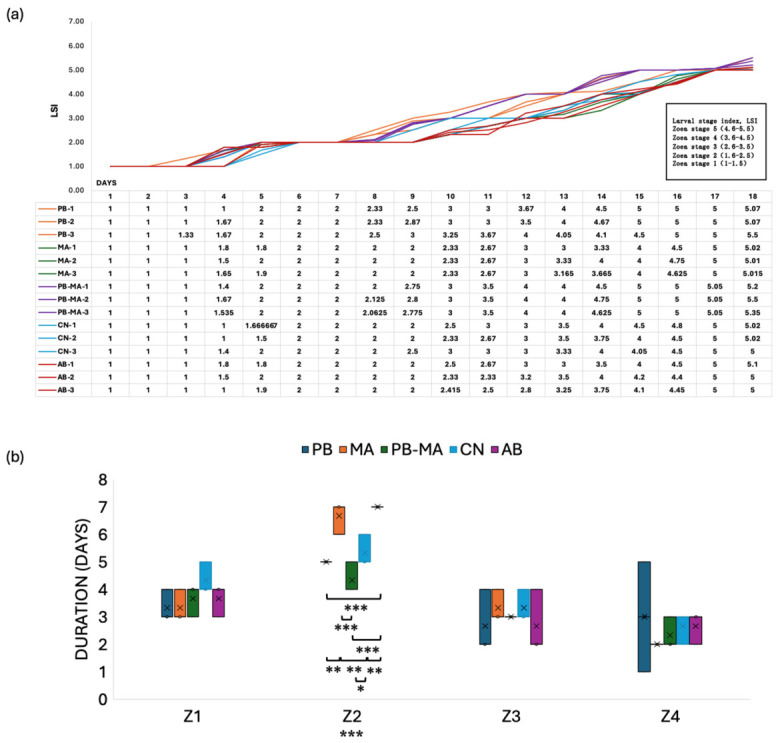
(**a**) Larval stage development and (**b**) duration of each zoea stage in *Scylla paramamosain* larviculture affected by different treatments. LSI, larval stage index; PB, probiotics alone; MA, microalgae alone; PB-MA, probiotics–microalgae consortium; CN, clear water control; AB, antibiotics alone. The samples were Z1–Z5 from different treatment groups. n = 12. * *p* < 0.05; ** *p* < 0.01; *** *p* < 0.001.

The developmental progression of *Scylla paramamosain* zoea is presented in Figure 2a. The PB-MA group exhibited the highest larval stage index (LSI), with developmental progression exceeding that of the control group on days 4, 9–16, and 18. This was followed by the PB group, which displayed accelerated development relative to the control group on days 8–16 and 18. The MA group showed faster developmental progression than the control group on days 4–5, while the AB group only exhibited accelerated development on day 5 and ultimately did not surpass the control group. Figure 2b compares the time intervals between successive larval molts. During the Z1 stage, all treatment groups exhibited an average reduction of 1 day compared to the control group. During the Z2 stage (LSI = 1.6–2.5), significant differences were observed among groups (*p* < 0.001), with the PB-MA group developing fastest, on average 3 days faster than the AB group and 2 days faster than the MA group (*p* < 0.001). No significant differences were detected among groups during the transition from Z3 to Z5. In terms of the total duration required for zoea larvae to complete metamorphosis to the Z5 stage, the PB-MA group required an average of 15 days, and the PB group 15.5 days, whereas the MA group, AB group, and control group each required 17 days.

### 2.2. Transcriptome Assembly and Unigene Annotation

Transcriptomic regulatory analysis was conducted via sequencing at two zoea stages exhibiting distinct larval morphology: Z1 and Z3. Although survival did not differ significantly among groups during the Z1 stage, transcriptomic profiling at this stage was retained because the Z1 phase represents the first exogenous feeding period and the initial interaction with microbiota reared in water. Early transcriptional reprogramming at Z1—such as the induction of transcription factors and stress-response genes—may establish molecular trajectories that later influence Z3-to-Z5 survival and developmental rate. Therefore, Z1 represents a unique window to capture the earliest host signals induced by different rearing environments, even in the absence of overt phenotypic divergence. A total of 60 cDNA libraries were constructed from samples collected at four developmental time points: Z1 at 6 h (initiation of metamorphosis), Z1 at 24 h (stable stage), Z3 at 6 h (initiation of metamorphosis), and Z3 at 24 h (stable stage). Following quality control, the sequencing yielded 90.51, 101.06, 103.08, and 96.24 Gb of clean data, respectively. The total number of unigenes assembled for the four developmental time points were 20,142, 20,194, 20,266, and 20,300, respectively. The Q30 base percentage for all samples was no less than 95.13%, indicating high sequencing data quality suitable for downstream analysis (Supplementary Table S1). NCBI BLAST+(v2.13.0) searches and functional annotation of the assembled unigenes were performed against seven public databases. The results showed that 20,142, 20,194, 20,266, and 20,300 assembled unigenes from the four transcriptomes were annotated in at least one database (Supplementary Table S2). Based on BLASTx alignments against the Nr database (which annotated 99% of the unigenes), the unigene sequences of *Scylla paramamosain* exhibited the highest similarity to those of *Penaeus vannamei* (62.87%, 62.66%, 62.74%, and 63.27%, respectively) (Supplementary Figure S1). Gene Ontology (GO) and Kyoto Encyclopedia of Genes and Genomes (KEGG) analyses were performed to elucidate the functional classification distribution of the annotated unigenes. In the GO classification, 5115, 5103, 5132, and 5146 unigenes were annotated at the four respective time points and categorized into 53 subgroups (Supplementary Figure S2). In the KEGG pathway enrichment analysis, 8764 unigenes at Z1 6 h were assigned to 267 pathways, 8762 unigenes at Z1 24 h were assigned to 272 pathways, 8780 unigenes at Z3 6 h were assigned to 273 pathways, and 8804 unigenes at Z3 24 h were assigned to 269 pathways.

### 2.3. Identification and Classification of Differentially Expressed Genes

**Figure 3:**
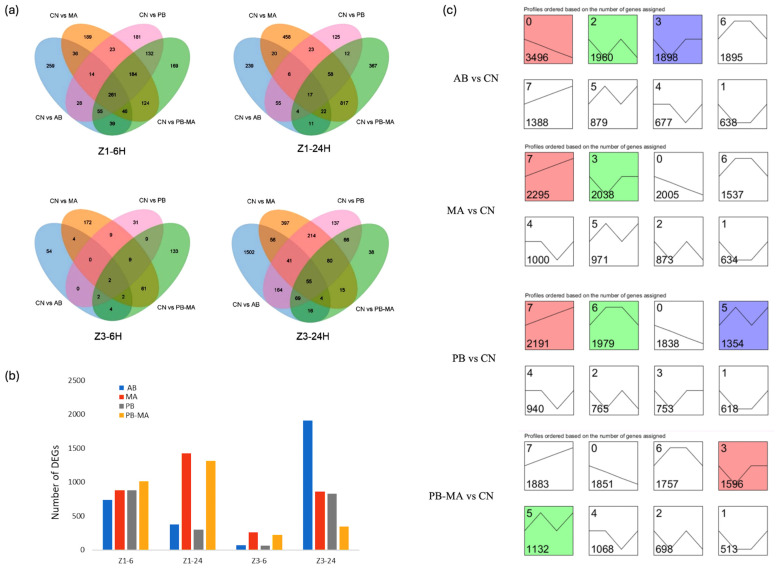
Profiles of differentially expressed genes presented as (**a**) Venn diagram, (**b**) bar chart and (**c**) gene cluster temporal profile, in *Scylla paramamosain* larviculture affected by different treatments at different developmental stages, including Z1 6 h, Z1 24 h, Z3 6 h, and Z3 24 h. PB, probiotics alone; MA, microalgae alone; PB-MA, probiotics–microalgae consortium; CN, clear water control; AB, antibiotics alone. DGEs, differentially expressed genes. Colored modules represent significantly enriched expression trend, while colorless modules indicate no significance.

During the Z1 and Z3 zoea stages, following 6 h and 24 h treatments with the four modulation regimes (AB, MA, PB, and PB-MA), transcriptome sequencing identified 1740, 2234, 492, and 2854 differentially expressed genes (DEGs) compared with the control, respectively (Figure 3a and Supplementary Table S3). Specifically, at 6 h of treatment during the Z1 stage, the AB, MA, PB, and PB-MA groups yielded 738, 877, 878, and 1010 DEGs, respectively. At 24 h of treatment during the Z1 stage, the groups yielded 374, 1421, 300, and 1308 DEGs, respectively. During the Z3 stage, at 6 h of treatment, the groups yielded 68, 259, 62, and 222 DEGs, respectively; at 24 h of treatment, the groups yielded 1907, 862, 826, and 343 DEGs, respectively (Figure 3b). The different modulation treatments exhibited distinct temporal trends in DEG numbers during the Z1 and Z3 zoea stages. At the 6 h time point in the Z1 stage, the number of DEGs identified across the different modulation groups was relatively comparable, and the number of shared DEGs across the four transcriptomes was the highest (261 DEGs). At the 6 h time point in the Z3 stage, all modulation groups exhibited the smallest numbers of DEGs, suggesting that Z3-stage zoeae may exhibit a certain lag in responsiveness to environmental modulation compared to the Z1 stage. The number of shared DEGs in the 24 h treatment groups was consistently lower than that in the 6 h groups, with pronounced differences observed among the treatment groups. Afterwards, the DEGs’ dynamic expression patterns of different treatments versus the control were clustered with the STEM (v1.3.13) software in Figure 3c. Temporal profiles were plotted in chronological order with colored modules representing significantly enriched gene expression trend modules, while colorless modules indicate no significance. PB-MA, PB, and MA presented significantly upregulated profiles (*p* ≤ 0.05), such as PB-MA profile 5 (1132 DEGs), PB profile 7 (2191 DEGs), and MA profile 7 (2295 DEGs). Whereas AB presented significantly downregulated profiles (*p* ≤ 0.05), such as AB profile 0 (3496 DEGs). GO enrichment analysis was performed on the DEGs identified from comparisons between each modulation group (AB, MA, PB, PB-MA) and the control group at different treatment time points (Z1-6 h, Z1-24 h, Z3-6 h, Z3-24 h). The results demonstrated that DEGs in all groups exhibited extremely significant enrichment levels (KS < 1 × 10^−30^) at each time point. At the biological process level, DEGs at Z1-6 h were primarily enriched in rapid stress-response pathways such as transcriptional regulation, protein modification, and basal phosphorus metabolism, including DNA-templated transcription (GO:0006351) and protein modification process (GO:0006464). At Z1-24 h, biological pathways associated with cell signal transduction, hormone regulation, and cellular stress responses were activated, indicating a marked increase in regulatory network complexity. At Z3-24 h, enrichment further extended to DNA repair, ion homeostasis maintenance, and immune defense processes. At the molecular function level, nucleic acid binding (GO:0003676) and DNA binding (GO:0003677) dominated across all groups and time points, with extremely significant enrichment. Additionally, protein kinase activity (GO:0004672), catalytic activity (GO:0003824), endopeptidase activity (GO:0004175), and transmembrane signaling receptor activity (GO:0004888) were consistently enriched at high abundance. At Z3-24 h, oxidoreductase activity (GO:0016491), ion channel activity, and phosphatase-related functions were further upregulated, ensuring a sustained activation of metabolic reactions and signal transduction.

### 2.4. DEGs Involved in Early Reacting Transcription Regulation

**Figure 4:**
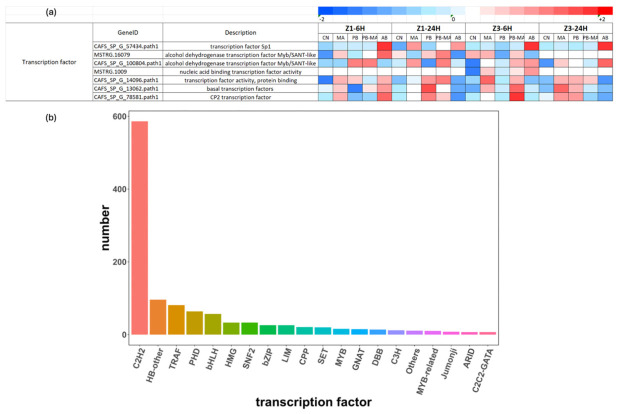
(**a**) Transcription factors that were significantly upregulated in Z1 6 h and Z3 6 h in *Scylla paramamosain* larviculture affected by different treatments at various time points, and (**b**) the number of amino acid sequences corresponding to different transcription factor families. CN, clear water control; MA, microalgae alone; PB, probiotics alone; PB-MA, probiotics–microalgae consortium; AB, antibiotics alone. The red color indicates the upregulated genes, and the blue color indicates the downregulated genes in the heatmap.

A total of 547 DEGs with transcription regulation functions in Z1 6 h and 571 DEGs with transcription regulation functions in Z3 6 h were commonly expressed in all treatment groups, as categorized in Figure 4b. Among these early-stage-reacting transcription factors (TFs) in the 6 h groups, significantly upregulated TFs (Fold Change ≥ 2 and *p* < 0.05) in the treatment groups versus the control are summarized in Figure 4a, including Sp1-like zinc finger proteins (SP1) belonging to the most dominant C2H2 zinc finger protein family, as shown in Figure 4b, Myb/SANT-domain transcription factors (MYB/SANT), and CP2 transcription factors (CP2). Based on the provided color-coded expression profiles across Z1 and Z3 at 6 h and 24 h, the majority of these TFs displayed differential regulation patterns. Specifically, the Myb/SANT-like TFs (CAFS_SP_G_100804.path1) exhibited upregulation for PB-MA in Z1 6 h, and the basal transcription factor (CAFS_SP_G_13062.path1) and CP2 transcription factor (CAFS_SP_G_78581.path1) exhibited upregulation for PB-MA in Z3 6 h, whereas the SP1 homolog (CAFS_SP_G_57434.path1) exhibited upregulation especially for AB in both Z1 and Z3 6 h. These TFs also exhibited upregulation in Z1 and/or Z3 24 h samples, indicating sustained transcriptional activation during late stress response. These findings imply that these early reacting TFs might play a prominent role in the long-term adaptation to different larviculture conditions in these samples.

### 2.5. DEGs Involved in Late Reacting KEGG Pathways

**Figure 5:**
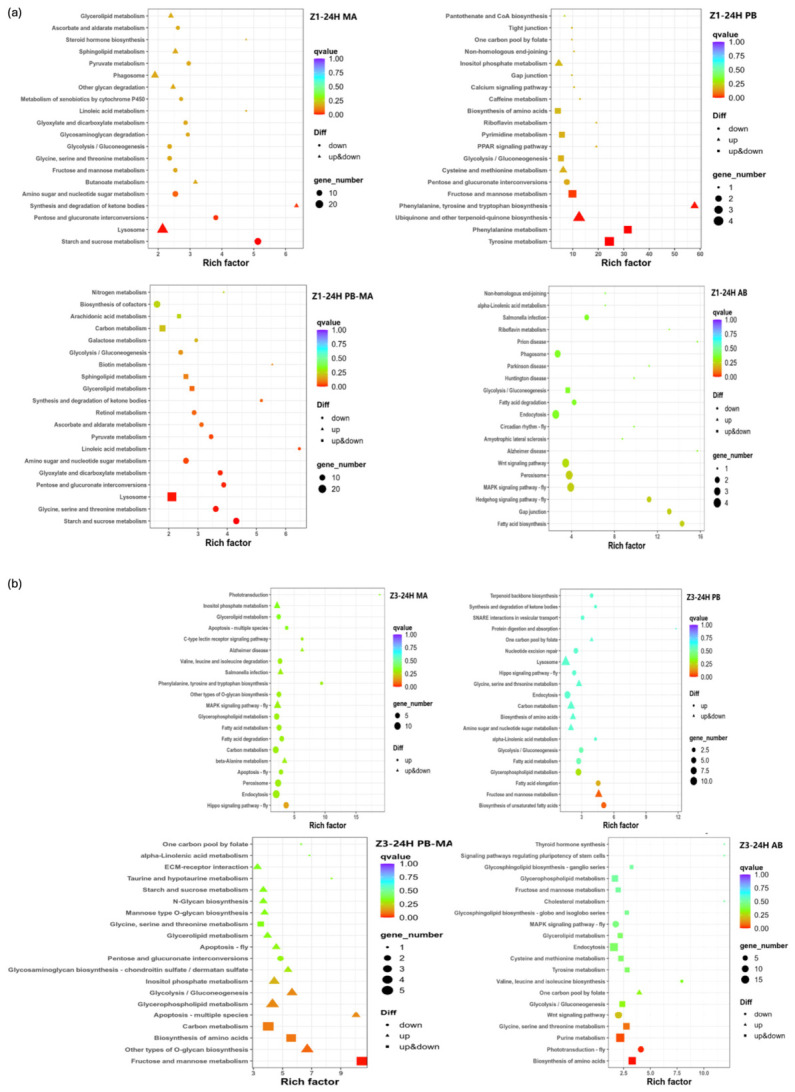
KEGG function classification of DEGs at (**a**) Z1 24 h and (**b**) Z3 24 h in *Scylla paramamosain* larviculture affected by different treatments versus control. PB, probiotics alone; MA, microalgae alone; PB-MA, probiotics–microalgae consortium; AB, antibiotics alone.

KEGG pathway analysis was performed on the 24 h groups of all treatments versus control (Figure 5) due to a more pronounced inter-treatment discrepancy in transcriptional response than that observed at 6 h. For Z1 24 h in Figure 5a, under MA treatment, lysosome (ko04142) and synthesis and degradation of ketone bodies (ko00072) were significantly enriched with both up- and down-regulated genes, while starch and sucrose metabolism (ko00500), pentose and glucuronate interconversions (ko00040), and amino sugar and nucleotide sugar metabolism (ko00520) were significantly enriched and generally down-regulated. Under PB treatment, significantly enriched pathways were Ubiquinone and other terpenoid-quinone biosynthesis (ko00130), phenylalanine, tyrosine, and tryptophan biosynthesis (ko00400), tyrosine metabolism (ko00350), phenylalanine metabolism (ko00360), and fructose and mannose metabolism (ko00051), most of which were up-regulated. Under PB-MA treatment, lysosome (ko04142) and Glycerolipid metabolism (ko00561) were significantly enriched and generally up-regulated, whereas starch and sucrose metabolism (ko00500), amino sugar and nucleotide sugar metabolism (ko00520), and Glycine, serine and threonine metabolism (ko00260) were also significantly enriched and continuously down-regulated. Under AB treatment, no statistically significant pathway was enriched; only fatty acid biosynthesis (ko00061) exhibited a high enrichment factor but was overall down-regulated with a small number of DEGs. At Z3-24 h in Figure 5b, under MA treatment, the Hippo signaling pathway–fly (ko04391) was significantly up-regulated, and endocytosis (ko04144), carbon metabolism (ko01200), and peroxisome (ko04146) showed an up-regulated tendency. Under PB treatment, highly enriched pathways included fructose and mannose metabolism (ko00051), biosynthesis of unsaturated fatty acids (ko01040), and fatty acid elongation (ko00062), which were predominantly up-regulated, and lysosome (ko04142) was universally upregulated. Under PB-MA treatment, most genes were up-regulated, and DEGs were significantly enriched in fructose and mannose metabolism (ko00051), glycerophospholipid metabolism (ko00564), other types of O-glycan biosynthesis (ko00514), and carbon metabolism (ko01200). Under AB treatment, biosynthesis of amino acids (ko01230) and purine metabolism (ko00230) were significantly enriched with divergent expression patterns, and phototransduction–fly (ko04745) was significantly down-regulated.

### 2.6. DEGs Involved in Immune-Related Pathways

**Figure 6:**
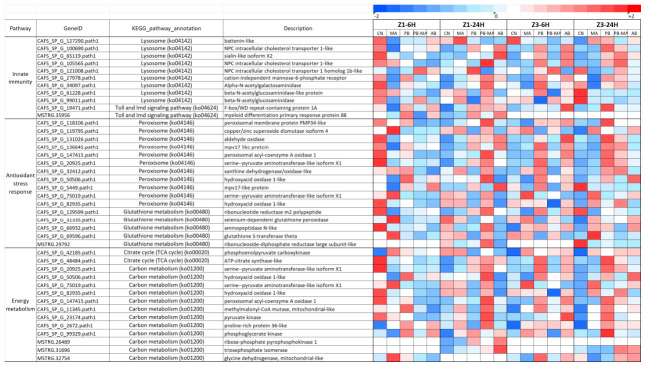
Commonly expressed differentially expressed genes related to different pathways in *Scylla paramamosain* larviculture affected by different treatments at various time points. CN, clear water control; MA, microalgae alone; PB, probiotics alone; PB-MA, probiotics–microalgae consortium; AB, antibiotics alone. The red color indicates the upregulated genes, and the blue color indicates the downregulated genes.

A total of 976 DEGs associated with immune-related gene expression were identified across the early (6 h) and late (24 h) time points in both Z1 and Z3 stages. PB-MA treatment generally induced milder transcriptional changes than AB exposure. Numerous genes encoding lysosomal hydrolases (e.g., hexosaminidases, glucosylceramidases, cathepsins), phagosome components, and pattern recognition receptors (Toll-like and NOD-like receptor pathways) were significantly downregulated in AB groups, whereas PB-MA maintained expression near control levels or even promoted upregulation. Pathway analysis further revealed that AB treatment suppressed key innate immunity modules, including phagosome maturation and lysosome-mediated degradation, whereas PB-MA preserved or enhanced these pathways. To precisely identify core differential pathways with biological significance, heatmap analysis of differentially expressed genes (DEGs) was performed across the different treatment groups within each time group (Figure 6). Inter-group comparisons primarily focused on two major immune pathways: the lysosome (ko04142) pathway and the Toll and Imd signaling pathway (ko04624). The results demonstrated that at Z1 6 h, genes in the lysosome pathway exhibited an overall downward trend, with most genes showing lower expression levels following treatment compared to the control group. Notably, the MA group exerted the most pronounced inhibitory effect on the lysosome pathway. The PB treatment significantly upregulated the cation-independent mannose-6-phosphate receptor in the lysosome pathway and myeloid differentiation primary response protein 88 (MyD88) in the Toll and Imd signaling pathway. The PB-MA treatment significantly induced the expression of NPC intracellular cholesterol transporter 1 homolog 1b-like in the lysosome pathway. At Z1 24 h, the overall expression levels of immune-related genes were higher than those in the control group, with most genes in the lysosome pathway and Toll and Imd signaling pathway being significantly upregulated. The PB-MA treatment exhibited the most effective activation of immune pathways, significantly enhancing the expression of signature lysosomal hydrolases such as NPC intracellular cholesterol transporter 1-like and Alpha-N-acetylgalactosaminidase. The MA group significantly upregulated battenin-like protein in the lysosome pathway. The PB group primarily activated NPC intracellular cholesterol transporter 1 homolog 1b-like in the lysosome pathway. The AB treatment upregulated a relatively smaller number of genes but specifically induced high expression of Sialin-like isoform X2 and NPC intracellular cholesterol transporter 1-like in the lysosome pathway. At the Z3 6 h stage, the majority of immune genes were significantly upregulated in the PB group, with the most prominent upregulation observed for Alpha-N-acetylgalactosaminidase in the lysosome pathway. The AB group exhibited the second-highest number of upregulated genes, with significant activation of F-box/WD repeat-containing protein 1A in the Toll and Imd signaling pathway. The PB-MA group significantly induced the expression of NPC intracellular cholesterol transporter 1 homolog 1b-like in the lysosome pathway. In contrast, the MA group displayed an overall suppressed gene expression state. At the Z3 24 h stage, most immune genes in both the PB and PB-MA groups exhibited an upward regulatory trend. Specifically, the PB group significantly upregulated NPC intracellular cholesterol transporter 1-like in the lysosome pathway and F-box/WD repeat-containing protein 1A in the Toll and Imd signaling pathway. The PB-MA group significantly activated NPC intracellular cholesterol transporter 1-like and Sialin-like isoform X2 in the lysosome pathway. The AB group, conversely, exhibited a generally suppressive state.

### 2.7. DEGs Involved in Oxidative Stress-Related Pathways

A comprehensive analysis of the transcriptomic data revealed a robust and intricate response of genes involved in maintaining cellular redox homeostasis. A total of 1185 DEGs associated with oxidative stress response were identified across all treatment comparisons. These genes were primarily enriched in the glutathione metabolism (ko00480), peroxisome (ko04146), and mitophagy–animal (ko04137) and predominantly enriched in two major pathways: peroxisome (ko04146) and glutathione metabolism (ko00480). The expression of oxidative stress-related pathway genes across treatment groups at different time points exhibited pronounced temporal and treatment-specific patterns (Figure 6). The results demonstrated that at the Z1 6 h stage, the MA group exhibited the highest number of upregulated genes. Within the peroxisome pathway, copper/zinc superoxide dismutase isoform 4 and serine–pyruvate aminotransferase-like isoform X1 were significantly upregulated, along with selenium-dependent glutathione peroxidase and glutathione S-transferase theta in the glutathione metabolism pathway. The remaining three groups displayed relatively low overall expression levels, with the majority of oxidative stress-related genes appearing suppressed. At the Z1 24 h stage, oxidative stress-related pathway genes were broadly activated across all treatment groups. The PB-MA treatment exhibited the most pronounced activation effect, with most genes in the peroxisome (ko04146) and glutathione metabolism (ko00480) pathways being significantly upregulated. Notably, the most prominent upregulation was observed for peroxisomal acyl-coenzyme A oxidase 1 and hydroxyacid oxidase 1-like within the peroxisome pathway. At the Z3 6 h stage, the AB group significantly induced the upregulation of aldehyde oxidase in the peroxisome pathway, with the most prominent increase observed. Both the peroxisome (ko04146) and glutathione metabolism (ko00480) pathways were generally upregulated in this group. The PB group also showed a considerable number of upregulated genes. The MA group specifically activated serine–pyruvate aminotransferase-like isoform X1 in the peroxisome pathway. The PB-MA group displayed an overall expression trend similar to that of the control group, with a comparatively weaker activation effect. At the Z3 24 h stage, the PB group significantly upregulated eight genes within the peroxisome pathway, including peroxisomal membrane protein PMP34-like, aldehyde oxidase, mpv17-like protein, peroxisomal acyl-coenzyme A oxidase 1, serine–pyruvate aminotransferase-like isoform X1, and xanthine dehydrogenase/oxidase-like. The PB-MA group also exhibited a distinct upward trend in the peroxisome (ko04146) pathway. The MA group upregulated glutathione S-transferase theta in the glutathione metabolism (ko00480) pathway. In contrast, the AB group was primarily characterized by the suppression of oxidative stress responses.

### 2.8. DEGs Involved in Energy Metabolism-Related Pathways

A total of 148 DEGs associated with central carbon and energy metabolism were identified across all time points and treatments. These genes were primarily enriched in the TCA cycle, oxidative phosphorylation, and carbon metabolism pathways. Notably, the expression dynamics in the PB-MA group were characterized by the sustained upregulation of key metabolic enzymes, suggesting enhanced nutrient assimilation and energy production compared to the AB group, which exhibited a more pronounced downregulation of these pathways. The expression patterns of significantly DEGs related to energy metabolism pathways exhibited marked differences under the various modulation treatments (Figure 6). At Z1 6 h, in the PB group, genes related to hydroxyacid oxidase 1-like, methylmalonyl-CoA mutase mitochondrial-like, proline-rich protein 36-like, and phosphoglycerate kinase within the carbon metabolism (ko01200) pathways were significantly induced. The MA group significantly induced the expression of phosphoenolpyruvate carboxykinase in the citrate cycle (TCA cycle) (ko00020) pathway, as well as serine–pyruvate aminotransferase-like isoform X1 and glycine dehydrogenase mitochondrial-like in the carbon metabolism (ko01200) pathway. In contrast, the majority of relevant genes in the PB-MA and AB groups were suppressed. At Z1 24 h, most genes in the carbon metabolism (ko01200) and citrate cycle (TCA cycle) (ko00020) pathways were significantly induced in the PB-MA group. Among these, the upregulation of hydroxyacid oxidase 1-like, pyruvate kinase, and methylmalonyl-CoA mutase mitochondrial-like within carbon metabolism (ko01200) was particularly pronounced. The majority of genes in the MA group also exhibited an upward trend, whereas the PB and AB groups were predominantly characterized by downregulated gene expression. At Z3 6 h, energy metabolism-related genes were generally activated across all modulation conditions. Gene upregulation was most prominent in the PB group, characterized by a significantly elevated expression of ATP-citrate synthase-like in the citrate cycle (TCA cycle) (ko00020), as well as serine–pyruvate aminotransferase-like isoform X1, hydroxyacid oxidase 1-like, methylmalonyl-CoA mutase mitochondrial-like, and glycine dehydrogenase mitochondrial-like in carbon metabolism (ko01200). In the MA group, only serine–pyruvate aminotransferase-like isoform X1 in carbon metabolism (ko01200) was significantly upregulated. The PB-MA treatment group exhibited a relatively weak overall upregulation, with no significantly upregulated genes detected. The AB group displayed a considerable number of upregulated genes, among which pyruvate kinase in carbon metabolism was significantly upregulated. At Z3 24 h, energy metabolism-related genes were upregulated across all modulation groups. The upregulation effect was most pronounced in the PB group, with significant upregulation of multiple genes, including ATP-citrate synthase-like in the citrate cycle (TCA cycle) (ko00020) and phosphoglycerate kinase and pyruvate kinase in carbon metabolism (ko01200). This was followed by the PB-MA group, which exhibited significant upregulation of hydroxyacid oxidase 1-like and triosephosphate isomerase in carbon metabolism (ko01200). The MA and AB groups showed a weaker upregulation of energy metabolism genes: in the MA group, only serine–pyruvate aminotransferase-like isoform X1 in carbon metabolism (ko01200) was significantly upregulated, while in the AB group, only hydroxyacid oxidase 1-like in carbon metabolism (ko01200) showed significant upregulation. This pattern (Figure 6) strongly supports the hypothesis that the PB-MA environment provides better nutritional support via the citrate cycle (TCA cycle) (ko00020) and carbon metabolism (ko01200), compared with antibiotic treatment.

## 3. Discussion

Over the past decades, the combined application of microalgae and probiotics has been recognized as a critical determinant of larval development and survival in aquaculture hatcheries [41]. Despite this potential, optimized species-specific combinations and defined dosages remain a key focus for enhancing large-scale production. Evidence supported that our specific microalgae—*Isochrysis galbana* [42], *Pavlova* spp. [43], *Chlorella vulgaris* [44], *Nitzschia closterium* [45], *Platymonas helgolandica* [46], and *Phaeodactylum tricornutum* [47] can provide essential polyunsaturated fatty acids, immunomodulatory metabolites, and prebiotic-like substrates. Concurrently, our specific probiotics, such as *Lactococcus lactis* [48], photosynthetic *Ectothiorhodospira shaposhnikovii* [35], and actinomycete *Arthrobacter* sp. [49], can improve gut colonization, water quality, and stress tolerance in larviculture. Their synergistic action is exemplified by *Navicula* sp. fermented with *Lactobacillus sakei*, which upregulates systemic immune and gut-associated genes in gilthead seabream [50] and by Humboldt squid silage enriched with *Lactobacillus sakei* in Pacific red snappers with improved physiological resilience and disease resistance [51]. Likewise, the co-administration of *Bacillus subtilis* with *P. tricornutum* or *Tetraselmis chuii* enhances complement activity, respiratory burst, and phagocytosis, with microalgal digestion products supporting probiotic growth better than glucose [52]. In the present study, the administration of a probiotics–microalgae consortium (PB-MA) to *Scylla paramamosain* zoea resulted in a significant enhancement of both developmental progression and survival rates compared to groups receiving antibiotics (AB), microalgae alone (MA), or probiotics alone (PB). The growth performance data clearly indicated that the PB-MA group exhibited superior stage-specific survival from the Z3 to Z5 stages, achieving a mean survival rate of 34% (Figure 1), which was significantly higher than that of the control and AB groups (*p* < 0.01). Furthermore, the PB-MA treatment accelerated the total developmental duration required to reach the Z5 stage. Larvae in the PB-MA group completed metamorphosis to Z5 in an average of 15 days, whereas the MA, AB, and control groups each required 17 days (Figure 2). This reduction in molt interval, particularly during the Z2 stage, where the PB-MA group developed 3 days faster than the AB group (*p* < 0.001), underscores the synergistic effect of the bacterio-algae consortium on larval physiology. Practically, the reduction in total larval duration could translate into lower feed and labor costs, reduced risk of pathogen accumulation, and increased annual hatchery production cycles, thus substantially improving economic viability for commercial mud crab hatcheries. These findings are in accordance with those observed in *Anthocidaris crassipina*, where a combined diet of *Chaetoceros muelleri* and *Rhodopseudomonas palustris* markedly improved digestive enzyme activity and metamorphosis [41]. It is also noteworthy that the relatively higher variability in Z1–Z2 survival observed in the control (CN) group may reflect the inherent sensitivity of early-stage larvae to subtle differences in maternal provisioning, initial microbial exposure, and micro-scale water quality fluctuations during the transition from endogenous to exogenous feeding. Because Z1 larvae are particularly dependent on maternally derived nutrients and gut microbiota priming, even minor differences in hatching batch quality can contribute to survival variance. To mitigate these effects, we used larvae from the same broodstock female for each replicate set, maintained strict temperature and salinity control, and excluded the visibly damaged or slow-swimming larvae before experimental allocation. Nonetheless, future studies could consider incorporating larger numbers of biological replicates or broodstock donors, as well as continuous water quality monitoring, to further reduce noise in early-stage survival data.

The observed phenotypic advantages are further substantiated by the transcriptional responses of the zoea. Recent transcriptomic evidence in aquatic larvae has demonstrated that antibiotic exposure generally suppresses immune and metabolic pathways [53,54], whereas probiotics activate innate immunity and stress-resilience signaling [55,56]. In the present study, differential gene expression analysis revealed distinct temporal regulation patterns among the treatment groups. During the Z1 stage, the PB-MA group yielded 1010 and 1308 differentially expressed genes (DEGs) at 6 h and 24 h post-treatment, respectively, representing a broader transcriptional remodeling than the MA or PB groups alone (Figure 3). Notably, STEM clustering analysis revealed significantly upregulated temporal profiles in the PB-MA (profile 5, 1132 DEGs), PB (profile 7, 2191 DEGs), and MA (Profile 7, 2295 DEGs) groups (*p* ≤ 0.05). In stark contrast, the AB group exhibited a significantly downregulated profile (profile 0, 3496 DEGs; *p* ≤ 0.05). This shift from suppressive transcriptional regulation under antibiotic exposure to activation under the PB-MA consortium suggests that the bacterio-algae assemblage fine-tunes the host transcriptome to enhance developmental progression and survival. Collectively, these results highlight that the PB-MA combination strategy offers a promising avenue for improving the health and productivity of crustacean larvae in commercial hatcheries.

Early reacting transcription factors (TFs) subsequently regulate lineage-specific genes and enhancers, and thus activate the zygotic genome after fertilization, guiding cell fate transitions during development [57]. In our study, a total of 547 and 571 DEGs with transcription regulation functions were commonly expressed across all treatment groups at Z1 6 h and Z3 6 h, respectively. Among these early-stage-reacting TFs (Figure 4), the Myb/SANT-like TF exhibited upregulation in PB-MA at Z1 6 h, while the basal transcription factor and CP2 TF were upregulated in PB-MA at Z3 6 h. In contrast, the SP1 homolog exhibited upregulation specifically in AB at both Z1 and Z3 6 h. The sustained upregulation observed in Z1 and/or Z3 24 h samples indicated prolonged transcriptional activation during the late stress response. The roles of these TFs in development and stress adaptation have been brought to light in previous studies. For instance, SP1 is a critical regulator of larval development, and its activity is modulated by antibiotics such as mithramycin, which specifically inhibits SP1 binding to GC-rich promoters [58,59]. Moreover, mithramycin reduces Sp1-dependent transcription of genes involved in cell proliferation and stress responses, potentially affecting larval survival pathways [60,61]. Furthermore, MYB/SANT factors maintain pluripotency during early differentiation [62,63], and CP2 factors such as TFCP2L1 are pivotal for embryonic stem cell self-renewal [64]. Together, these findings imply that the differential regulation of SP1, MYB/SANT, and CP2 TFs may represent a key transcriptional nexus through which distinct larviculture environments modulate early developmental pathways and long-term adaptation in these samples.

Lysosome-dependent degradation and Toll/IMD signaling constitute fundamental pillars of crustacean innate immunity, orchestrating both cellular homeostasis and humoral defense against environmental perturbations [65,66]. In our study, comparative transcriptomic analysis across Z1 and Z3 zoeal stages revealed distinct regulatory patterns in these pathways following exposure to PB, MA, PB-MA, and AB. At the early Z1 6 h time point, the lysosome pathway (ko04142) exhibited a general downward trend relative to the control (Figure 6); however, PB treatment uniquely upregulated the cation-independent mannose-6-phosphate receptor, suggesting a specific enhancement of lysosomal enzyme trafficking. Notably, the PB-MA treatment significantly induced the expression of NPC intracellular cholesterol transporter 1 homolog 1b-like (*NPC1*), a critical mediator of lysosomal cholesterol egress whose dysfunction is linked to severe lipid storage disorders [67,68]. By Z1 24 h, a pronounced activation of immune pathways was observed, with PB-MA treatment most effectively upregulating signature lysosomal hydrolases, including *NPC1* and Alpha-N-acetylgalactosaminidase (*NAGA*). Given that *NAGA* deficiency results in pathological glycoconjugate accumulation and neurodegeneration [69,70], its elevated expression underscores the potential of PB-MA to fortify lysosomal catabolic capacity. Conversely, AB treatment, while inducing a smaller subset of genes, specifically promoted a high expression of Sialin-like isoform X2 (*Sialin*), a transporter essential for sialic acid export whose impairment causes lysosomal free sialic acid storage disorders [71,72]. At the Z3 stage, the PB group prominently upregulated *NAGA* and *NPC1*, whereas the PB-MA group maintained the activation of *Sialin* and *NPC1*, indicating a sustained enhancement of endolysosomal function. Within the Toll and Imd signaling pathway (ko04624), PB treatment significantly induced MyD88 at Z1 6 h, and both PB and PB-MA groups robustly activated F-box/WD repeat-containing protein 1A (*FBXW1A*) at Z3 24 h. *FBXW1A*, encoding the substrate recognition component of the SCF E3 ubiquitin ligase complex, is known to modulate protein stability under cellular stress [73,74]. In stark contrast, AB exposure frequently suppressed phagosome maturation and lysosome-mediated degradation, aligning with previous evidence in insects comparing probiotic administration with antibiotic-induced dysbiosis that altered Toll/IMD transcriptional profiles and compromised antimicrobial peptide synthesis [75,76]. Collectively, these findings demonstrate that while AB treatment perturbs innate immune homeostasis, the PB-MA consortium preserves and often amplifies the expression of key lysosomal hydrolases and pattern recognition signaling components. The specific upregulation of *NPC1*, *NAGA*, and *Sialin* suggests that enhanced cholesterol trafficking and glycoconjugate catabolism may be associated with improved immune resilience, although further functional validation is required to establish direct protective roles in the context of aquaculture pathogen challenges.

Peroxisome function and glutathione metabolism constitute essential detoxification and redox homeostasis modules in aquatic invertebrates, equipping organisms with resilience against diverse environmental and xenobiotic stressors [77,78]. In our study, a comprehensive transcriptomic survey across Z1 and Z3 zoeal stages following exposure to MA, PB, PB-MA, and AB identified 1185 DEGs associated with oxidative stress responses, which were predominantly enriched in the peroxisome (ko04146) and glutathione metabolism (ko00480) pathways (Figure 6). At the Z1 6 h stage, the MA group exhibited the most pronounced activation, with significant upregulation of copper/zinc superoxide dismutase isoform 4 and serine–pyruvate aminotransferase-like isoform X1 within the peroxisome pathway, alongside selenium-dependent glutathione peroxidase and glutathione S-transferase theta in glutathione metabolism, suggesting an early mobilization of antioxidant defenses. By Z1 24 h, oxidative stress-related pathways were broadly activated across all treatments; however, the PB-MA group demonstrated the most robust response, notably inducing peroxisomal acyl-coenzyme A oxidase 1 (*ACOX1*) and hydroxyacid oxidase 1-like (*HAO1*). *ACOX1* encodes the rate-limiting enzyme of peroxisomal fatty acid β-oxidation and is transcriptionally regulated by PPARα, serving as a sensitive biomarker of xenobiotic challenge and a target for probiotic-mediated metabolic restoration [79,80]. Meanwhile, *HAO1* is a peroxisomal flavoenzyme whose substrate glycolate accumulates following antibiotic-induced gut dysbiosis, linking its expression to metabolic perturbation [81]. At the Z3 6 h stage, the AB group significantly induced aldehyde oxidase (*AOX*), a molybdo-flavoenzyme implicated in phase I xenobiotic oxidation and insecticide resistance [82], yet broader suppression characterized the AB group at later time points. In contrast, at Z3 24 h, the PB group prominently upregulated eight peroxisomal genes, including peroxisomal membrane protein PMP34-like, *AOX*, mpv17-like protein, *ACOX1*, serine–pyruvate aminotransferase-like isoform X1, and xanthine dehydrogenase/oxidase-like, reflecting a sustained enhancement of peroxisomal biogenesis and catabolic capacity. The PB-MA group similarly exhibited a distinct upward regulatory trend in the peroxisome pathway. Notably, antibiotic exposure has been documented to dysregulate glutathione-dependent redox defense by depleting GSH and inhibiting GST activity, while concurrently impairing peroxisome function through PPAR downregulation in the swimming crab *Portunus trituberculatus* larvae [83] and in zebrafish *Danio rerio* larvae [84]. Conversely, probiotic supplementation counteracts such toxicity by restoring GSH levels and upregulating PPARβ-driven fatty acid catabolism [85,86]. Collectively, our findings demonstrate that PB-MA co-administration preserves and often amplifies the expression of core peroxisomal β-oxidation enzymes and glutathione-dependent antioxidants, whereas AB exposure disrupts this delicate redox balance. The specific induction of *ACOX1* and *HAO1* in the PB-MA group suggests that enhanced peroxisomal lipid catabolism and glyoxylate metabolism may underpin improved oxidative stress resilience in crustacean larvae, although further functional characterization is required to establish direct protective roles during pathogen or pollutant challenges.

Carbon metabolism (ko01200) and the citrate cycle (TCA cycle, ko00020) are fundamental to larval energy homeostasis, integrating glycolysis, gluconeogenesis, and aerobic respiration to supply both ATP and biosynthetic precursors essential for rapid growth and successful metamorphosis [87]. In our study, a total of 148 DEGs associated with central carbon and energy metabolism were identified across the Z1 and Z3 zoeal stages following exposure to MA, PB, PB-MA, and AB. At the Z1 6 h stage, the PB group significantly induced the expression of *HAO1*, methylmalonyl-CoA mutase mitochondrial-like (*MUT*), and phosphoglycerate kinase within carbon metabolism (ko01200), while the MA group prominently upregulated phosphoenolpyruvate carboxykinase in the TCA cycle (ko00020) and serine–pyruvate aminotransferase-like isoform X1 in carbon metabolism. Notably, *MUT* encodes a mitochondrial enzyme that converts methylmalonyl-CoA to succinyl-CoA, linking branched-chain amino acid catabolism to the TCA cycle; deficiency of *MUT* in *Drosophila* larvae results in mitochondrial dysfunction and impaired mitophagy, underscoring its critical role in metabolic homeostasis [88]. In contrast, the PB-MA and AB groups exhibited general suppression of these pathways at this early time point. By Z1 24 h, the PB-MA group demonstrated the most robust activation, with pronounced upregulation of *HAO1*, pyruvate kinase (*PK*), and *MUT* within carbon metabolism. *PK* catalyzes the terminal step of glycolysis, and its loss in *Drosophila* larvae disrupts glycolytic flux and induces compensatory shifts in lipid metabolism [89], whereas xenobiotic stress, such as chlorbenzuron exposure, significantly enhances *PK* activity and mRNA expression, leading to perturbed energy metabolism [90,91]. At the Z3 6 h stage, energy metabolism-related genes were broadly activated, with the PB group exhibiting the most prominent upregulation, including ATP-citrate synthase-like in the TCA cycle and *HAO1*, *MUT*, and glycine dehydrogenase mitochondrial-like in carbon metabolism. The AB group also displayed a considerable number of upregulated genes, notably *PK* in carbon metabolism. At Z3 24 h, the PB group maintained a strong upregulation signature, significantly inducing ATP-citrate synthase-like, phosphoglycerate kinase, and *PK*. The PB-MA group followed, with significant induction of *HAO1* and triosephosphate isomerase (*TPI*). *TPI* is a critical glycolytic enzyme whose deficiency impairs synaptic vesicle recycling and causes neurological deficits in *Drosophila* larvae [92], and its expression can be downregulated by antibiotic exposure, as observed in *Enterococcus faecium* under vancomycin stress [93]. Collectively, these findings demonstrate that PB and PB-MA treatments promote sustained upregulation of key glycolytic and TCA cycle enzymes, suggesting enhanced nutrient assimilation and energy production capacity. In contrast, the AB group exhibited a more variable and often suppressed metabolic profile, aligning with evidence that antibiotic exposure can alter glycolytic enzyme abundance and disrupt carbohydrate metabolism [94]. The specific induction of *MUT*, *PK*, and *TPI* in probiotic-supplemented groups indicates that the modulation of carbon metabolism and the TCA cycle flux may serve as a candidate mechanism for improved larval fitness and stress resilience, although further functional validation is required to establish direct causal relationships in crustacean developmental contexts.

**Figure 7:**
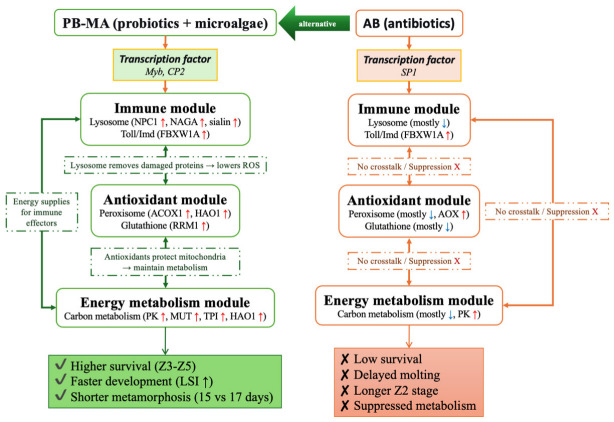
Hypothetical model illustrating the contrasting regulatory networks in *Scylla paramamosain* zoea under PB-MA (probiotics + microalgae) versus AB (antibiotics) treatments. The red arrows indicate upregulation; the blue indicates downregulation. SP1, transcription factor SP1; FBXW1A, F-box/WD repeat-containing protein 1A; AOX, aldehyde oxidase; PK, pyruvate kinase; ROS, reactive oxygen species; Myb, alcohol dehydrogenase transcription factor Myb/SANT-like; CP2, transcription factor CP2; NPC1, NPC intracellular cholesterol transporter 1 homolog 1b-like; NAGA, Alpha-N-acetylgalactosaminidase; Sialin, Sialin-like isoform X2; ACOX1, peroxisomal acyl-coenzyme A oxidase 1; HAO1, hydroxyacid oxidase 1-like; RRM1, ribonucleoside-diphosphate reductase large subunit-like; MUT, methylmalonyl-CoA mutase mitochondrial-like; TPI, triosephosphate isomerase. ↑, upregulation; ↓, downregulation; →, consequence; X, suppression; ✓, advantage; ✗, disadvantage.

In our study, we found that *HAO1* was uniquely elevated in both oxidative stress-related pathways and energy metabolism-related pathways, suggesting a sophisticated crosstalk between peroxisomal redox regulation and central carbon flux. For example, at the Z1 24 h stage in the PB-MA treatment group, peroxisomal acyl-coenzyme A oxidase 1 and *HAO1* were significantly upregulated within the peroxisome pathway (ko04146), while key carbon metabolism genes such as pyruvate kinase and methylmalonyl-CoA mutase mitochondrial-like were concurrently induced in the citrate cycle (TCA cycle) (ko00020) and carbon metabolism (ko01200) pathways (Figure 2). This could be illustrated by previous research showing that *HAO1* is a peroxisomal flavoenzyme generating H_2_O_2_ that modulates cellular redox balance and can drive specific cellular responses [95]. In addition, in plants, the homologous glycolate oxidase similarly produces photorespiratory H_2_O_2_ in peroxisomes, integrating metabolism with stress signaling [96]. Therefore, the sustained upregulation of *HAO1* observed under the PB-MA environment may closely relate to enhanced nutrient assimilation and energy production, demonstrating that peroxisomal function directly cross-regulates mitochondrial carbon flux and redox homeostasis during larval development. Mechanistically, in Figure 7, PB-MA combinations maintain lysosomal integrity, thereby sustaining energy metabolism and reducing oxidative damage in larval tissues. This lysosome-centered pathway prevents apoptosis and preserves cellular homeostasis under rearing stress. It is also possible that PB-MA maintains a healthier microbiota and facilitates nutrient absorption. Consequently, PB-MA crosstalk orchestrates a multi-targeted defense—enhancing nutrient assimilation, immune resilience, and oxidative balance—to accelerate larval development and survival. To be specific, sustained lysosomal activity and Toll/Imd signaling maintain immune surveillance, while enhanced peroxisomal β-oxidation and glutathione metabolism buffer oxidative stress, and the upregulation of glycolytic and TCA cycle flux ensures sufficient energy supply for growth and molting. This integrated response, summarized in Figure 7, positions the lysosome as a central hub linking immunity, redox balance, and energy metabolism under PB-MA treatment. Therefore, the strategic integration of probiotic bacteria and microalgae offers a robust, multifactorial approach to sustainable larviculture without the selective pressures and homeostasis disruptions inherent to traditional chemotherapeutic interventions.

Given their consistent and treatment-specific regulation patterns, these genes—particularly *NPC1*, *NAGA*, *ACOX1*, and *MUT*—may serve as candidate transcriptomic biomarkers for assessing larval immune competence and metabolic status in hatchery production. Routine qPCR screening of these targets could provide hatchery operators with early indicators of physiological resilience, enabling timely adjustment of rearing protocols. The core molecular pathways identified here—lysosome, Toll/Imd signaling, peroxisomal β-oxidation, glutathione metabolism, and TCA cycle—are highly conserved across decapod crustaceans and share significant overlap with teleost innate immunity. Consequently, the consortium-based strategy validated in *S. paramamosain* holds considerable promise for adaptation to other commercially important crustaceans, such as *Penaeus vannamei* and *Eriocheir sinensis*, and potentially marine finfish larviculture. The transcriptomic markers identified here—particularly *NPC1*, *ACOX1*, and *MUT*—could serve as quantitative readouts in high-throughput screening platforms to rationally select and optimize probiotic–microalgal combinations for maximal immune and metabolic benefit in target species. Future work integrating proteomics and targeted metabolomics will be essential to validate the protein-level and metabolite-level consequences of the transcriptional signatures reported here and to build a holistic model of larval physiological resilience under probiotic–microalgal rearing regimes.

We acknowledge several limitations of this study. First, while the transcriptomic data provide extensive mechanistic insights, they were not independently validated at the protein or metabolite level; future multi-omics approaches are warranted. Second, the experimental system, although carefully controlled, does not fully replicate the complexity of large-scale commercial hatcheries, where feed variation, microbial dynamics, and operator effects introduce additional variability. Finally, the study was limited to a single mud crab species; testing the generality of the PB-MA strategy across phylogenetically diverse crustaceans will be essential for broader industry adoption.

Beyond the direct benefits to larval fitness, replacing antibiotics with probiotic–microalgal consortia addresses a critical One Health concern. Reduced antibiotic input mitigates selective pressure for antimicrobial resistance genes in hatchery microbiomes and surrounding coastal waters, thereby limiting horizontal gene transfer to human-pathogenic and environmental bacteria. Industry adoption of such microbial management strategies could therefore contribute to global antimicrobial stewardship goals while simultaneously enhancing production sustainability.

## 4. Materials and Methods

### 4.1. Ethics Statement

All animal procedures performed in this investigation conformed to the applicable national and international regulations. The experimental protocol was reviewed and approved by the East China Sea Fisheries Research Institute. Collecting wild mud crabs from marine waters in China does not require special permits. No endangered or legally protected species were utilized. We employed the minimum necessary number of crab larvae to avoid any impact on the next generation’s breeding stock, and larval harm was reduced by immediate euthanasia after sampling.

### 4.2. Study Animals

This experiment was conducted in June 2023 at the Zhejiang Ninghai Experimental Base of the East China Sea Fisheries Research Institute in Ninghai Town, Zhejiang Province, China (29.2° N, 121.5° E). Water treatment was performed as in our previous study [34]. Only female, uninjured egg-bearing mud crabs (*Scylla paramamosain*) with the same hatching date were selected. The newly hatched larvae by different mother crabs were immediately transferred to separate 80-cubic-meter seedling ponds [34]. The nursery pool was maintained as in the previous study [34]. The zoea of the mud crab can be divided into five developmental stages: zoea (Z) in stages I (Z1), II (Z2), III (Z3), IV (Z4), and V (Z5), when Z1 larvae are characterized by a rounded carapace with simple rostrum and sessile compound eyes, and they rely primarily on yolk reserves and early exogenous feeding. Z2 larvae develop stalked eyes and more pronounced abdominal segmentation. Z3 exhibits lengthened rostral spines and pleopod buds. Z4 and Z5 larvae show progressively more developed pleopods and telson structures, with Z5 being the final zoeal stage before metamorphosis to megalopa.

### 4.3. Experimental Design and Sampling

Larvae hatched by the same mother crab were randomly assigned to five treatment groups, with three batches of larvae hatched by three different mother crabs selected as three biological replicates for all treatment groups. Previous studies on *Scylla serrata* larvae [97] and *Scylla paramamosain* larvae [34] ensure a sufficient sample size (150,000 zoea I) for further analysis.

Five experimental groups were established via daily water supplementation: clear water control with no additions (CN), microalgae alone (MA), probiotics alone (PB), consortium with both microalgae and probiotics (PB-MA), and antibiotic group (AB). All groups received identical live prey without enrichment, ensuring sole evaluation of water-borne supplementation effects. The commercial probiotic mixture used was purchased from Jiangsu Hengtai Environmental Protection Technology Development Co., Ltd. (Wuxi, China), at pH 3.0–4.0, with a lactic acid bacteria count of 1.0 × 10^7^–1.0 × 10^8^ CFU/mL, a yeast count of 1.0 × 10^4^–1.0 × 10^5^ CFU/mL, a photosynthetic bacteria count of 1.0 × 10^3^–2.0 × 10^3^ CFU/mL, and an actinomycete count of 1.0 × 10^3^–3.0 × 10^3^ CFU/mL, with over 80 viable bacterial species and over 10% metabolites. The instantaneous probiotics concentration in water reached 10^6^ CFU/mL [35] and was able to inhibit harmful bacteria, improve water quality, promote nutrient absorption and immunity of larvae, and maintain the balance of bacteria and algae. Microalgae treatment contained six beneficial microalgae strains identified from the mud crab larviculture, as in Patent No. CN117502329B by our unpublished study, *Isochrysis galbana*, *Pavlova viridis*, *Chlorella vulgaris*, *Nitzschia closterium f. minutissima*, *Platymonas subcordiformis*, and *Phaeodactylum tricornutum*, which were cultured in-house using a modified nutrient medium containing ferric citrate, NH_4_Cl, sodium silicate, and KH_2_PO_4_ at 25–30 °C under 6000–7000 lx illumination. Microalgal cultures were cultivated to densities exceeding 10^5^ ind/L before use. Final target concentrations in the rearing water were maintained at 10^5^ ind/L total microalgae biomass [98] with a biodiversity index of >1.12, achieved through daily addition. These algae provide essential PUFAs, enrich live prey, regulate water color, and dissolve oxygen, and together establish a stable ecological algal phase for indoor larviculture. The probiotics–microalgae consortium was prepared by mixing the commercial probiotic mixture with the self-cultured dense microalgae suspensions prior to application, as in Patent No. CN117502329B. The commercial probiotic mixture comprises *Lactococcus lactis* (predominant lactic acid bacterium), *Saccharomyces cerevisiae*, *Ectothiorhodospira shaposhnikovii* (photosynthetic bacterium), and *Arthrobacter* sp. (actinomycete), originally isolated from healthy mud crab larvae reared in water and selected for their demonstrated antagonism toward *Vibrio* spp., water-quality improvement capacity, and immunomodulatory properties. The microalgal consortium includes six strains—*Isochrysis galbana*, *Pavlova viridis*, *Chlorella vulgaris*, *Nitzschia closterium f. minutissima*, *Platymonas subcordiformis*, and *Phaeodactylum tricornutum*—all originally isolated from mud crab hatchery tanks, characterized by high PUFA content and broadly overlapping cell size ranges suitable for zoeal ingestion. The antibiotic used was 10 ppm florfenicol as described in our previous study [34].

Z1 and Z3 are two key developmental stages of *Scylla paramamosain* zoea with significant differences in morphology [99]. At 6 h (initiation of metamorphosis) and 24 h (stable stage) after the initiation of the zoea stage, Z1 and Z3, 100 zoea larvae were randomly collected from each replicate. After residual seawater was removed by vacuum filtration, a total of 60 zoea samples with three replicates in five treatment groups at four time points were immediately transferred into 2 mL RNase-free centrifuge tubes containing RNA preservation solution for immediate transcription analysis as previously described [33] and in Section 4.6.

### 4.4. Growth Performance Measurement

For each treatment, four 5 L beakers were collected daily after dusk from each of the three replicate rearing tanks, providing 12 replicate samples for counting the larvae [34]. From these counts, survival rates and the larval stage index (LSI) were determined. Because the number of larvae removed by daily sampling was negligible relative to the tank population, it did not appreciably affect the pond-level survival estimates.

Survival rate: Stage-specific survival was calculated as the percentage of successfully molted zoea that were alive at dusk on the first day of that stage, compared with the initial Z1 count projected from the beaker samples.

Larval stage index (LSI): Each larva in the beaker was examined under a microscope to assign a developmental score (Z1 = 1, Z2 = 2, etc.) [100]. Daily LSI was then computed using the formula: LSI = ((A1 × A2) + (B1 × B2))/C, where A1 is the number of larvae still in the preceding stage, A2 is the score of the preceding stage, B1 is the number of larvae in the current developmental stage, B2 is the score of the current stage, and C is the total number of larvae inspected.

### 4.5. Statistical Analysis

All statistical analyses for growth performance were performed using IBM SPSS Statistics V26.0 (IBM Corporation, Armonk, NY, USA). Normality and homogeneity of variances were confirmed using Shapiro–Wilk and Levene’s tests, respectively. Stage-specific survival rates and larval stage index (LSI) data were compared among treatment groups using one-way analysis of variance (ANOVA) followed by Fisher’s least significant difference (LSD) post hoc test. The time intervals between successive larval molts were also compared by one-way ANOVA with LSD correction. A significance level of *p* < 0.05 was used for all phenotypic comparisons. The exact *p*-values were categorized as follows: *p* < 0.05 denoted significant differences, *p* < 0.01 denoted highly significant differences, and *p* < 0.001 denoted very highly significant differences.

### 4.6. Total RNA Isolation and Sequencing

*Scylla paramamosain* zoea total RNA was isolated using a total RNA extraction Kit (TIANGEN Biotech Co., Ltd., Beijing, China), followed by DNase I treatment to remove the genomic DNA contamination. RNA concentration and purity were determined using a NanoDrop 2000 spectrophotometer (Agilent Technologies, Santa Clara, CA, USA). RNA integrity was assessed using the RNA Nano 6000 Assay Kit with an Agilent 2100 Bioanalyzer system (Agilent Technologies, Santa Clara, CA, USA). DNA nanoballs were generated by library construction using the Hieff NGS^®^ mRNA Isolation Master Kit (Yeasen Biotechnology Co., Ltd., Shanghai, China). After library quality validation and quantification by Qubit, sequencing was carried out on the MGI DNBSEQ-T7 platform (MGI Tech Co., Ltd., Shenzhen, China). The prepared DNBs were loaded onto a microarray chip, and sequencing was performed using combinatorial probe anchor synthesis (cPAS) technology to generate raw sequencing data.

### 4.7. Gene Sequence Alignment and Functional Annotation

Raw sequencing reads in FASTQ v0.11.9 format were processed using in-house Perl scripts. Specifically, reads containing adapter sequences, ambiguous poly-N bases, and low-quality reads were filtered out to generate high-quality clean reads. Data quality was evaluated based on Q20, Q30, GC content, and sequence duplication levels. Sequence alignment was performed between the clean reads of the 60 samples and the designated reference genome of *Scylla paramamosain,* reported previously [101] using Hisat2 v2.2.1 [102], with only reads showing perfect matches or a single mismatch retained for further analyses and annotation. Gene functional annotation was performed using multiple public databases, including Nr (NCBI non-redundant protein sequences) [103], Nt (NCBI non-redundant nucleotide sequences), Pfam (Protein family) [104], KOG/COG (Clusters of Orthologous Groups of proteins), Swiss-Prot (manually annotated and reviewed protein sequences, KO (KEGG Orthology database), and GO (Gene Ontology).

### 4.8. Analysis of Differentially Expressed Genes

Gene expression levels, including those of known genes and novel genes, were quantified based on the read alignment results using the StringTie v2.2.1 software [105]. The expression abundance of each gene was calculated as FPKM (fragments per kilobase of transcript per million fragments mapped) to eliminate the influences of gene length and sequencing depth [106]. Differentially expressed genes (DEGs) between treatments with biological replicates were identified using the DESeq2 v1.38.0 R package [107]. The fold change (FC) was defined as the ratio of expression levels between the two groups. The resulting *p*-values were adjusted using the Benjamini–Hochberg procedure [108] to control the false discovery rate (FDR). Genes with FDR < 0.05 and |log_2_(FC)| > 1 were considered as significantly differentially expressed and were classified as upregulated or downregulated according to their expression patterns. Gene Ontology (GO) enrichment analysis was conducted using the GOseq v1.50.0 R package [109], which accounts for gene length bias in the enrichment tests. The Kyoto Encyclopedia of Genes and Genomes (KEGG) database was used to annotate and analyze high-level functions of the biological systems, and the KOBAS v3.0 software was employed to identify significantly enriched KEGG pathways of the DEGs [110]. Based on GO annotations and the Pfam database, category analysis and heatmap construction were performed for transcription factor (TF) related functional genes encoded by DEGs that were significantly upregulated at 6 h in Z1 and Z3 under different treatments. The aim was to focus on the early activated TFs in different treatments.

### 4.9. DEGs Temporal Expression Patterns Analysis

DEGs of zoea larvae Z1 and Z3 at 6 h and 24 h under different treatments were analyzed using the Short Time-series Expression Miner (STEM) v1.3.13 software [111] to compare the temporal expression patterns between treatments and control using log_2_FC to control as the expression level and identify statistically significant profiles in color at *p* < 0.05.

### 4.10. Gene Expression Heatmap Analysis

To visualize the expression patterns of DEGs in Z1 and Z3 at 6 h and 24 h after different treatments, expression heatmap analysis was performed. According to the FPKM values of DEGs in different treatment groups at the same time point, significant upregulated genes involved in immune regulation, oxidative stress, and energy metabolism pathways were screened from the KEGG enrichment results. The expression heatmaps of key DEGs in these critical KEGG pathways were constructed using R 4.4.3.

## 5. Conclusions

In this study, five rearing treatment groups (control, microalgae alone, probiotics alone, probiotics–microalgae consortium, and antibiotic) were established, and transcriptomic sequencing was performed at 6 and 24 h post-treatment in the first and third zoeal stages of *Scylla paramamosain* larvae. A better physiological performance, including significantly higher stage-specific survival rates and faster larval development, was observed in the probiotics–microalgae consortium group than in the control, antibiotic, and probiotics-alone groups. For transcriptome results, a dynamic DEGs analysis showed a discrepant profile from transcription regulation, immune-related pathways, oxidative stress-related pathways, and energy metabolism-related pathways across treatment groups. Moreover, some candidate genes (e.g., *NPC1*, *NAGA*, *Sialin*, *ACOX1*, *HAO1*, *MUT*, *PK*, *TPI*) were highlighted in our study, and their roles in enhancing larval resilience in crustacean aquaculture need to be further functionally validated. Overall, our results systematically elucidate, for the first time, the molecular mechanisms by which a probiotics–microalgae consortium enhances basal immunity, antioxidant defenses, and metabolic adaptability in mud crab larvae, which is shown in Figure 7, and also provide solid molecular evidence and a theoretical foundation for developing effective antibiotic alternatives in sustainable aquaculture.

## 6. Patents

There is a patent resulting from the work reported in this manuscript: Yin Fu et al., “A method for constructing an ecological algae phase for efficient indoor mud crab seedling rearing”, CN117502329B (issued 17 February 2026).

## Data Availability

The original contributions presented in this study are included in the article. Further inquiries can be directed to the corresponding author.

## Supplementary Materials

The following supporting information can be downloaded at https://www.mdpi.com/article/10.3390/antibiotics15050498/s1. Table S1: Sequencing statistics: The *Scylla paramamosain* larviculture was affected by different treatments at different developmental stages, including Z1 6 h, Z1 24 h, Z3 6 h, and Z3 24 h. PB, probiotics alone; MA, microalgae alone; PB-MA, probiotics–microalgae consortium; CN, clear water control; AB, antibiotics alone. Table S2: Function annotation statistics: The *Scylla paramamosain* larviculture was affected by different treatments at different developmental stages, including Z1 6 h, Z1 24 h, Z3 6 h, and Z3 24 h. PB, probiotics alone; MA, microalgae alone; PB-MA, probiotics–microalgae consortium; CN, clear water control; AB, antibiotics alone. Figure S1: Species distribution in NR annotation: The *Scylla paramamosain* larviculture was affected by different treatments at different developmental stages, including Z1 6 h, Z1 24 h, Z3 6 h, and Z3 24 h. Figure S2: GO classification: The *Scylla paramamosain* larviculture was affected by different treatments at different developmental stages, including Z1 6 h, Z1 24 h, Z3 6 h, and Z3 24 h. Table S3: Statistics of differentially expressed genes: The *Scylla paramamosain* larviculture was affected by different treatments at different developmental stages, including Z1 6 h, Z1 24 h, Z3 6 h, and Z3 24 h. PB, probiotics alone; MA, microalgae alone; PB-MA, probiotics–microalgae consortium; CN, clear water control; AB, antibiotics alone. DGE, differentially expressed gene.

## References

[1] Vadstein O., Attramadal K.J.K., Bakke I., Olsen Y. (2018). K-Selection as Microbial Community Management Strategy: A Method for Improved Viability of Larvae in Aquaculture. Front. Microbiol..

[2] Leal J.F., Henriques I.S., Correia A., Santos E.B.H., Esteves V.I. (2017). Antibacterial activity of oxytetracycline photoproducts in marine aquaculture’s water. Environ. Pollut..

[3] Guo X., Chen H., Tong Y., Wu X., Tang C., Qin X., Guo J., Li P., Wang Z., Liu W. (2024). A review on the antibiotic florfenicol: Occurrence, environmental fate, effects, and health risks. Environ. Res..

[4] Wang Q., Xu Z., Wang Y., Huo G., Zhang X., Li J., Hua C., Li S., Zhou F. (2023). Transcriptomics Analysis of the Toxicological Impact of Enrofloxacin in an Aquatic Environment on the Chinese Mitten Crab (*Eriocheir sinensis*). Int. J. Environ. Res. Public Health.

[5] Yuan X., Lv Z., Zhang Z., Han Y., Liu Z., Zhang H. (2023). A Review of Antibiotics, Antibiotic Resistant Bacteria, and Resistance Genes in Aquaculture: Occurrence, Contamination, and Transmission. Toxics.

[6] Gutiérrez-Pacheco M.M., Gracia-Valenzuela M.H., Ortega-Ramirez L.A., Vázquez-Armenta F.J., Leyva J.M., Ayala-Zavala J.F., Chávez-Almanza A.F. (2026). Joining Forces Against Antibiotic Resistance in Aquaculture: The Synergism Between Natural Compounds and Antibiotics. Antibiotics.

[7] Luo K., Yang Z., Wen X., Wang D., Liu J., Wang L., Fan R., Tian X. (2024). Recovery of intestinal microbial community in Penaeus vannamei after florfenicol perturbation. J. Hazard. Mater..

[8] Bardhan A., Abraham T.J., Dash G., Nagesh T.S., Sau S.K., Patil P.K. (2024). Intestinal Histopathological Aberrations in Oreochromis niloticus Juveniles upon Dietary Florfenicol Administration. Bull. Environ. Contam. Toxicol..

[9] Vieco-Saiz N., Belguesmia Y., Raspoet R., Auclair E., Gancel F., Kempf I., Drider D. (2019). Benefits and Inputs From Lactic Acid Bacteria and Their Bacteriocins as Alternatives to Antibiotic Growth Promoters During Food-Animal Production. Front. Microbiol..

[10] Kari Z.A., Téllez-Isaías G., Khoo M.I., Wee W., Kabir M.A., Cheadoloh R., Wei L.S. (2024). Resveratrol impacts on aquatic animals: A review. Fish Physiol. Biochem..

[11] Lin X.L., Kang Z.W., Pan Q.J., Liu T.X. (2015). Evaluation of five antibiotics on larval gut bacterial diversity of *Plutella xylostella* (Lepidoptera: Plutellidae). Insect Sci..

[12] Defoirdt T., Sorgeloos P., Bossier P. (2011). Alternatives to antibiotics for the control of bacterial disease in aquaculture. Curr. Opin. Microbiol..

[13] Vine N.G., Leukes W.D., Kaiser H. (2006). Probiotics in marine larviculture. FEMS Microbiol. Rev..

[14] Contente D., Díaz-Formoso L., Feito J., Gómez-Sala B., Costas D., Hernández P.E., Muñoz-Atienza E., Borrero J., Poeta P., Cintas L.M. (2024). Antimicrobial Activity, Genetic Relatedness, and Safety Assessment of Potential Probiotic Lactic Acid Bacteria Isolated from a Rearing Tank of Rotifers (*Brachionus plicatilis*) Used as Live Feed in Fish Larviculture. Animals.

[15] Sonnenschein E.C., Phippen C.B.W., Bentzon-Tilia M., Rasmussen S.A., Nielsen K.F., Gram L. (2018). Phylogenetic distribution of roseobacticides in the *Roseobacter* group and their effect on microalgae. Environ. Microbiol. Rep..

[16] Grotkjær T., Bentzon-Tilia M., D’Alvise P., Dourala N., Nielsen K.F., Gram L. (2016). Isolation of TDA-producing *Phaeobacter* strains from sea bass larval rearing units and their probiotic effect against pathogenic *Vibrio* spp. in Artemia cultures. Syst. Appl. Microbiol..

[17] Schaeck M., Duchateau L., Van den Broeck W., Van Trappen S., De Vos P., Coulombet C., Boon N., Haesebrouck F., Decostere A. (2016). *Vibrio lentus* protects gnotobiotic sea bass (*Dicentrarchus labrax* L.) larvae against challenge with *Vibrio harveyi*. Vet. Microbiol..

[18] Dos Santos T.M., Pereira L.A.B., Dos Santos L.P.C., Lopes A.L., Sampaio L.A.F., de Matos Silva G.V.G., Ferreira J.N., Dos Reis G.T.J., Teixeira J.B., Diniz-Neto R.J.T. (2026). Prospection of Autochthonous Lactic Acid Bacteria Displaying Probiotic Potential to Enhance *Colossoma macropomum* Larvae Growth and Immunoprotection Against *Aeromonas hydrophila*. J. Fish Dis..

[19] Contente D., Díaz-Formoso L., Feito J., Hernández P.E., Muñoz-Atienza E., Borrero J., Poeta P., Cintas L.M. (2024). Genomic and Functional Evaluation of Two *Lacticaseibacillus paracasei* and Two *Lactiplantibacillus plantarum* Strains, Isolated from a Rearing Tank of Rotifers (*Brachionus plicatilis*), as Probiotics for Aquaculture. Genes.

[20] Goulden E.F., Hall M.R., Pereg L.L., Baillie B.K., Høj L. (2013). Probiont niche specialization contributes to additive protection against *Vibrio owensii* in spiny lobster larvae. Environ. Microbiol. Rep..

[21] Nhan D.T., Cam D.T., Wille M., Defoirdt T., Bossier P., Sorgeloos P. (2010). Quorum quenching bacteria protect Macrobrachium rosenbergii larvae from *Vibrio harveyi* infection. J. Appl. Microbiol..

[22] Tarnecki A.M., Wafapoor M., Phillips R.N., Rhody N.R. (2019). Benefits of a *Bacillus* probiotic to larval fish survival and transport stress resistance. Sci. Rep..

[23] Yıldırım Ş., Suzer C., Fırat K., Saka Ş., Hekimoğlu M., Çoban D., Korkut A.Y., Köse İ., Antepli O., Gökvardar A. (2024). Impact of probiotic *Bacillus* sp. dietary supplementation on pancreatic and intestinal activities in seabream *Sparus aurata*. Lett. Appl. Microbiol..

[24] Hemaiswarya S., Raja R., Ravi Kumar R., Ganesan V., Anbazhagan C. (2011). Microalgae: A sustainable feed source for aquaculture. World J. Microbiol. Biotechnol..

[25] Morris H.J., Carrillo O., Almarales A., Bermúdez R.C., Lebeque Y., Fontaine R., Llauradó G., Beltrán Y. (2007). Immunostimulant activity of an enzymatic protein hydrolysate from green microalga *Chlorella vulgaris* on undernourished mice. Enzym. Microb. Technol..

[26] Chakraborty R.D., Chakraborty K., Radhakrishnan E.V. (2007). Variation in fatty acid composition of *Artemia salina* nauplii enriched with microalgae and baker’s yeast for use in larviculture. J. Agric. Food Chem..

[27] Patil V., Källqvist T., Olsen E., Vogt G., Gislerød H.R. (2007). Fatty acid composition of 12 microalgae for possible use in aquaculture feed. Aquac. Int..

[28] Sivakumar K., Kannappan S., Vijayakumar B. (2023). Docking Studies on Biomolecules from Marine Microalga Skeletonema costatum Against Hemolysin Protein of Bioluminescence Disease-Causing *Vibrio harveyi*. Curr. Microbiol..

[29] Cerezuela R., Guardiola F.A., Meseguer J., Esteban M.Á. (2012). Enrichment of gilthead seabream (*Sparus aurata* L.) diet with microalgae: Effects on the immune system. Fish Physiol. Biochem..

[30] Maftuch M., Toban M.H., Risjani Y. (2012). Administration of marine algae (*Gracilaria verrucosa*) immunostimulant enhances some innate immune parameters in black tiger shrimp (*Penaeus monodon* Fabricus) against *Vibrio harveyi* infection. J. Appl. Sci. Res..

[31] Landolt M.L. (1989). The relationship between diet and the immune response of fish. Aquaculture.

[32] D’Alvise P.W., Lillebø S., Prol-Garcia M.J., Wergeland H.I., Nielsen K.F., Bergh Ø., Gram L. (2012). *Phaeobacter gallaeciensis* reduces *Vibrio anguillarum* in cultures of microalgae and rotifers, and prevents vibriosis in cod larvae. PLoS ONE.

[33] Fu Y., Zhang F., Wang W., Xu J., Zhao M., Ma C., Cheng Y., Chen W., Su Z., Lv X. (2024). Temporal and Spatial Signatures of *Scylla paramamosain* Transcriptome Reveal Mechanistic Insights into Endogenous Ovarian Maturation under Risk of Starvation. Int. J. Mol. Sci..

[34] Fu Y., Cheng Y., Ma L., Zhou Q. (2024). Longitudinal Microbiome Investigations Reveal Core and Growth-Associated Bacteria During Early Life Stages of *Scylla paramamosain*. Microorganisms.

[35] Wen C.Q., Xue M., Liang H.F., Wu Y., Li X. (2015). Beneficial effects of *Ectothiorhodospira shaposhnikovii* WF on larval cultivation of *Litopenaeus vannamei*. Benef. Microbes.

[36] Nishikawa A., Tsunoda K., Hatano K., Tabuchi Y., Hirano T., Izumi R., Oshima S., Furusawa Y., Sakatoku A., Srivastav A.K. (2025). Acute toxicity of local anesthetics used for humans and other animals in the larvae of marine crustacean, red-clawed crab *Chiromantes haematocheir*. J. Vet. Med. Sci..

[37] Powell D., Knibb W., Remilton C., Elizur A. (2015). De-novo transcriptome analysis of the banana shrimp (*Fenneropenaeus merguiensis*) and identification of genes associated with reproduction and development. Mar. Genom..

[38] Farhadi A., Tang S., Huang M., Yu Q., Xu C., Li E. (2023). Identification of key immune and stress related genes and pathways by comparative analysis of the gene expression profile under multiple environmental stressors in pacific white shrimp (*Litopenaeus vannamei*). Fish Shellfish Immunol..

[39] Yang Y., Jin F., Liu W., Huo G., Zhou F., Yan J., Zhou K., Li P. (2023). Comparative transcriptome, digital gene expression and proteome profiling analyses provide insights into the brachyurization from the megalopa to the first juvenile in *Eriocheir sinensis*. Heliyon.

[40] Wu Q., Cui W., Jiang C., Sun L., Feng Q., Su F. (2026). Hematopoietic and immune homeostasis programs of the Polian vesicle after evisceration in *Apostichopus japonicus*. Comp. Biochem. Physiol. Part A Mol. Integr. Physiol..

[41] Chu T.W., Chu Y., Sun W.T., Pan C.Y., Pan C.H., Ding D.S. (2024). Nutrient enrichment and probiotics for sea urchin *Anthocidaris crassipina* larvae in captivity to promote large-scale aquaculture. J. Anim. Physiol. Anim. Nutr..

[42] Huanacuni J.I., Pepe-Victoriano R., Lora-Vilchis M.C., Merino G.E., Torres-Taipe F.G., Espinoza-Ramos L.A. (2021). Influence of Microalgae Diets on the Biological and Growth Parameters of *Oithona nana* (Copepoda: Cyclopoida). Animals.

[43] Garcia A.S., Parrish C.C., Brown J.A. (2008). Growth and lipid composition of Atlantic cod (*Gadus morhua*) larvae in response to differently enriched *Artemia franciscana*. Fish Physiol. Biochem..

[44] Taufik M., Adnan A.S., Bolong Abol Munafi A., Mohd Noor N.A., Shahrul I., Ikhwanuddin M. (2022). Microalgal Preference and Feeding Density of Selected Microalgae Diets by Blue Swimming Crab *Portunus pelagicus* (Linnaeus, 1758). Pak. J. Biol. Sci..

[45] Hunt J., Birch G., Warne M.S., Krassoi R. (2009). Direct toxicity assessment of volatile chlorinated hydrocarbon-contaminated groundwater and derivation of a site-specific guideline. Integr. Environ. Assess. Manag..

[46] Liao K., Chen W., Zhang R., Zhou H., Xu J., Zhou C., Yan X. (2017). qPCR analysis of bivalve larvae feeding preferences when grazing on mixed microalgal diets. PLoS ONE.

[47] Bravo-Tello K., Ehrenfeld N., Solís C.J., Ulloa P.E., Hedrera M., Pizarro-Guajardo M., Paredes-Sabja D., Feijóo C.G. (2017). Effect of microalgae on intestinal inflammation triggered by soybean meal and bacterial infection in zebrafish. PLoS ONE.

[48] Loh J.Y., Kay G.L., Ting A.S.Y. (2018). Bioencapsulation and Colonization Characteristics of Lactococcus lactis subsp. lactis CF4MRS in *Artemia franciscana*: A Biological Approach for the Control of Edwardsiellosis in Larviculture. Mar. Biotechnol..

[49] Lauzon H.L., Gudmundsdottir S., Steinarsson A., Oddgeirsson M., Petursdottir S.K., Reynisson E., Bjornsdottir R., Gudmundsdottir B.K. (2010). Effects of bacterial treatment at early stages of Atlantic cod (*Gadus morhua* L.) on larval survival and development. J. Appl. Microbiol..

[50] Reyes-Becerril M., Guardiola F., Rojas M., Ascencio-Valle F., Esteban M. (2013). Dietary administration of microalgae *Navicula* sp. affects immune status and gene expression of gilthead seabream (*Sparus aurata*). Fish Shellfish Immunol..

[51] Reyes-Becerril M., Ascencio-Valle F., Macias M.E., Maldonado M., Rojas M., Esteban M.Á. (2012). Effects of marine silages enriched with Lactobacillus sakei 5-4 on haemato-immunological and growth response in Pacific red snapper (*Lutjanus peru*) exposed to Aeromonas veronii. Fish Shellfish Immunol..

[52] Cerezuela R., Guardiola F.A., González P., Meseguer J., Esteban M.Á. (2012). Effects of dietary *Bacillus subtilis*, *Tetraselmis chuii*, and *Phaeodactylum tricornutum*, singularly or in combination, on the immune response and disease resistance of sea bream (*Sparus aurata* L.). Fish Shellfish Immunol..

[53] Pereiro P., Rey-Campos M., Figueras A., Novoa B. (2022). An environmentally relevant concentration of antibiotics impairs the immune system of zebrafish (*Danio rerio*) and increases susceptibility to virus infection. Front. Immunol..

[54] Xiaowen C., Jiahao L., Zhaorun D., Wenfeng L., Richou H., Yanping C., Huichun X., Yi Z. (2024). Honeybee symbiont *Bombella apis* could restore larval-to-pupal transition disrupted by antibiotic treatment. J. Insect Physiol..

[55] Hesser J., Mueller R.S., Langdon C., Schubiger C.B. (2024). Immunomodulatory effects of a probiotic combination treatment to improve the survival of Pacific oyster (*Crassostrea gigas*) larvae against infection by *Vibrio coralliilyticus*. Front. Immunol..

[56] Xia Y., Cao J., Wang M., Lu M., Chen G., Gao F., Liu Z., Zhang D., Ke X., Yi M. (2019). Effects of *Lactococcus lactis* subsp. lactis JCM5805 on colonization dynamics of gut microbiota and regulation of immunity in early ontogenetic stages of tilapia. Fish Shellfish Immunol..

[57] Barral A., Zaret K.S. (2024). Pioneer factors: Roles and their regulation in development. Trends Genet..

[58] Czyz M., Gniazdowski M. (1998). Actinomycin D specifically inhibits the interaction between transcription factor Sp1 and its binding site. Acta Biochim. Pol..

[59] Kormanec J., Novakova R., Csolleiova D., Feckova L., Rezuchova B., Sevcikova B., Homerova D. (2020). The antitumor antibiotic mithramycin: New advanced approaches in modification and production. Appl. Microbiol. Biotechnol..

[60] Previdi S., Malek A., Albertini V., Riva C., Capella C., Broggini M., Carbone G.M., Rohr J., Catapano C.V. (2010). Inhibition of Sp1-dependent transcription and antitumor activity of the new aureolic acid analogues mithramycin SDK and SK in human ovarian cancer xenografts. Gynecol. Oncol..

[61] Schweer D., McCorkle J.R., Rohr J., Tsodikov O.V., Ueland F., Kolesar J. (2021). Mithramycin and Analogs for Overcoming Cisplatin Resistance in Ovarian Cancer. Biomedicines.

[62] van den Boom V., Kooistra S.M., Boesjes M., Geverts B., Houtsmuller A.B., Monzen K., Komuro I., Essers J., Drenth-Diephuis L.J., Eggen B.J. (2007). UTF1 is a chromatin-associated protein involved in ES cell differentiation. J. Cell Biol..

[63] Amiard S., Feit L., Vanrobays E., Simon L., Le Goff S., Loizeau L., Wolff L., Butter F., Bourbousse C., Barneche F. (2024). The TELOMERE REPEAT BINDING proteins TRB4 and TRB5 function as transcriptional activators of PRC2-controlled genes to regulate plant development. Plant Commun..

[64] Kotarba G., Krzywinska E., Grabowska A.I., Taracha A., Wilanowski T. (2018). TFCP2/TFCP2L1/UBP1 transcription factors in cancer. Cancer Lett..

[65] Lu Y., Liu Y., Cao J., Zhang Y., Zheng Y., Wang F. (2025). Waterborne ammonia toxicity damages crustacean hemocytes via lysosome-dependent autophagy: A case study of swimming crabs *Portunus trituberculatus*. Environ. Res..

[66] Chen D., Wang H. (2022). Redclaw crayfish (*Cherax quadricarinatus*) responds to *Vibrio parahaemolyticus* infection by activating toll and immune deficiency signaling pathways and transcription of associated immune response genes. Fish Shellfish Immunol..

[67] Long T., Qi X., Hassan A., Liang Q., De Brabander J.K., Li X. (2020). Structural basis for itraconazole-mediated NPC1 inhibition. Nat. Commun..

[68] Li X., Wang J., Coutavas E., Shi H., Hao Q., Blobel G. (2016). Structure of human Niemann-Pick C1 protein. Proc. Natl. Acad. Sci. USA.

[69] Desnick R.J., Wang A.M. (1990). Schindler disease: An inherited neuroaxonal dystrophy due to alpha-N-acetylgalactosaminidase deficiency. J. Inherit. Metab. Dis..

[70] Michalski J.C., Klein A. (1999). Glycoprotein lysosomal storage disorders: Alpha- and beta-mannosidosis, fucosidosis and alpha-N-acetylgalactosaminidase deficiency. Biochim. Biophys. Acta.

[71] Winchester B.G. (2001). Lysosomal membrane proteins. Eur. J. Paediatr. Neurol..

[72] Prolo L.M., Vogel H., Reimer R.J. (2009). The lysosomal sialic acid transporter sialin is required for normal CNS myelination. J. Neurosci..

[73] Fuhrmann D.C., Wittig I., Dröse S., Schmid T., Dehne N., Brüne B. (2018). Degradation of the mitochondrial complex I assembly factor TMEM126B under chronic hypoxia. Cell. Mol. Life Sci..

[74] Deng W., Vanderbilt D.B., Lin C.C., Martin K.H., Brundage K.M., Ruppert J.M. (2015). SOX9 inhibits β-TrCP-mediated protein degradation to promote nuclear GLI1 expression and cancer stem cell properties. J. Cell Sci..

[75] Guarnieri A., Falcone M., Brancazio N., Mukhtar F., Worku A.T., Cutuli M.A., Iacovino V.P., Ganassi S., De Cristofaro A., Di Marco R. (2025). In vivo functional screening on innate immunity of lactic acid bacteria in *Galleria mellonella* preclinical model: Comparative analysis of *Lactiplantibacillus plantarum* and *Lentilactobacillus kefiri*. Front. Cell. Infect. Microbiol..

[76] Sarvari M., Mikani A., Mehrabadi M. (2020). The innate immune gene Relish and Caudal jointly contribute to the gut immune homeostasis by regulating antimicrobial peptides in *Galleria mellonella*. Dev. Comp. Immunol..

[77] Orbea A., Ortiz-Zarragoitia M., Solé M., Porte C., Cajaraville M.P. (2002). Antioxidant enzymes and peroxisome proliferation in relation to contaminant body burdens of PAHs and PCBs in bivalve molluscs, crabs and fish from the Urdaibai and Plentzia estuaries (Bay of Biscay). Aquat. Toxicol..

[78] Liu Z., Zhang Y., Zheng Y., Feng Y., Zhang W., Gong S., Lin H., Gao P., Zhang H. (2023). Genome-wide identification glutathione-S-transferase gene superfamily in *Daphnia pulex* and its transcriptional response to nanoplastics. Int. J. Biol. Macromol..

[79] Li M., Wang Y., Tang Z., Wang H., Hu J., Bao Z., Hu X. (2022). Expression Plasticity of Peroxisomal Acyl-Coenzyme A Oxidase Genes Implies Their Involvement in Redox Regulation in Scallops Exposed to PST-Producing Alexandrium. Mar. Drugs.

[80] Weng H., Endo K., Li J., Kito N., Iwai N. (2015). Induction of peroxisomes by butyrate-producing probiotics. PLoS ONE.

[81] Uebanso T., Yoshimoto A., Aizawa S., Nakamura M., Masuda R., Shimohata T., Mawatari K., Takahashi A. (2020). Glycolate is a Novel Marker of Vitamin B(2) Deficiency Involved in Gut Microbe Metabolism in Mice. Nutrients.

[82] Coleman M., Vontas J.G., Hemingway J. (2002). Molecular characterization of the amplified aldehyde oxidase from insecticide resistant *Culex quinquefasciatus*. Eur. J. Biochem..

[83] Ren X., Wang Z., Gao B., Liu P., Li J. (2017). Toxic responses of swimming crab (*Portunus trituberculatus*) larvae exposed to environmentally realistic concentrations of oxytetracycline. Chemosphere.

[84] Lei Y., Li F., Mortimer M., Li Z., Peng B.X., Li M., Guo L.H., Zhuang G. (2023). Antibiotics disrupt lipid metabolism in zebrafish (*Danio rerio*) larvae and 3T3-L1 preadipocytes. Sci. Total Environ..

[85] Sun B., Liu M., Tang L., Hu C., Huang Z., Zhou X., Chen L. (2021). Probiotic supplementation mitigates the developmental toxicity of perfluorobutanesulfonate in zebrafish larvae. Sci. Total Environ..

[86] Avella M.A., Olivotto I., Silvi S., Place A.R., Carnevali O. (2010). Effect of dietary probiotics on clownfish: A molecular approach to define how lactic acid bacteria modulate development in a marine fish. Am. J. Physiol. Regul. Integr. Comp. Physiol..

[87] Wang C., An L., Dong X.S., Xu X., Feng X.Y., Wang Z.Z., He F., Chen X., Zhu Y.A., Meng Q.L. (2024). The tricarboxylic acid cycle is inhibited under acute stress from carbonate alkalinity in the gills of *Eriocheir sinensis*. Comp. Biochem. Physiol. Part D Genom. Proteom..

[88] Luciani A., Schumann A., Berquez M., Chen Z., Nieri D., Failli M., Debaix H., Festa B.P., Tokonami N., Raimondi A. (2020). Author Correction: Impaired mitophagy links mitochondrial disease to epithelial stress in methylmalonyl-CoA mutase deficiency. Nat. Commun..

[89] Heidarian Y., Tourigny J.P., Fasteen T.D., Mahmoudzadeh N.H., Hurlburt A.J., Nemkov T., Reisz J.A., D’Alessandro A., Tennessen J.M. (2023). Metabolomic analysis of *Drosophila melanogaster* larvae lacking pyruvate kinase. G3 Genes Genomes Genet..

[90] Zhao Y., Zou C., Zhang L., Li C., Li X., Song L. (2023). Chlorbenzuron caused growth arrest through interference of glycolysis and energy metabolism in *Hyphantria cunea* (Lepidoptera: Erebidae) larvae. Pestic. Biochem. Physiol..

[91] Zhang S., Zhang Y., Zou H., Li X., Zou H., Wang Z., Zou C. (2023). FDP-Na-induced enhancement of glycolysis impacts larval growth and development and chitin biosynthesis in fall webworm, *Hyphantria cunea* (Lepidoptera: Arctiidae). Pestic. Biochem. Physiol..

[92] Stone A., Cujic O., Rowlett A., Aderhold S., Savage E., Graham B., Steinert J.R. (2023). Triose-phosphate isomerase deficiency is associated with a dysregulation of synaptic vesicle recycling in *Drosophila melanogaster*. Front. Synaptic Neurosci..

[93] Ramos S., Chafsey I., Silva N., Hébraud M., Santos H., Capelo-Martinez J.L., Poeta P., Igrejas G. (2015). Effect of vancomycin on the proteome of the multiresistant *Enterococcus faecium* SU18 strain. J. Proteom..

[94] Veloso Lde C., dos Santos K.V., de Andrade H.M., Pires Sda F., dos Santos S.G., Vaz Trindade M.J., de Farias Lde M., de Carvalho M.A. (2013). Proteomic changes in Bacteroides fragilis exposed to subinhibitory concentration of piperacillin/tazobactam. Anaerobe.

[95] Peng X., Zhou Y., Chen J., Wang Z., Peng X., Wang H., Shu L., Liu N., Long Y., Linghu L. (2026). Peroxisome-derived and liver-specific hydroxyacid oxidase 1-mediated hydrogen peroxide promotes ferroptosis by augmenting peroxisomal ROS. J. Adv. Res..

[96] Li X., Chen L., Zeng X., Wu K., Huang J., Liao M., Xi Y., Zhu G., Zeng X., Hou X. (2023). Wounding induces a peroxisomal H_2_O_2_ decrease via glycolate oxidase-catalase switch dependent on glutamate receptor-like channel-supported Ca^2^+ signaling in plants. Plant J..

[97] Suprayudi M.A., Takeuchi T., Hamasaki K. (2012). Phospholipids Effect on Survival and Molting Synchronicity of Larvae Mud Crab *Scylla serrata*. HAYATI J. Biosci..

[98] Pires A. (2024). Laboratory and Field Culture of Larvae of The Slipper Limpet, *Crepidula fornicata*. J. Vis. Exp..

[99] Syafaat M.N., Azra M.N., Waiho K., Fazhan H., Abol-Munafi A.B., Ishak S.D., Syahnon M., Ghazali A., Ma H., Ikhwanuddin M. (2021). A Review of the Nursery Culture of Mud Crabs, Genus *Scylla*: Current Progress and Future Directions. Animals.

[100] Sui L., Wille M., Cheng Y., Sorgeloos P. (2007). The effect of dietary n-3 HUFA levels and DHA/EPA ratios on growth, survival and osmotic stress tolerance of Chinese mitten crab *Eriocheir sinensis* larvae. Aquaculture.

[101] Zhao M., Wang W., Zhang F., Ma C., Liu Z., Yang M.H., Chen W., Li Q., Cui M., Jiang K. (2021). A chromosome-level genome of the mud crab (*Scylla paramamosain* estampador) provides insights into the evolution of chemical and light perception in this crustacean. Mol. Ecol. Resour..

[102] Kim D., Langmead B., Salzberg S.L. (2015). HISAT: A fast spliced aligner with low memory requirements. Nat. Methods.

[103] Deng Y.Y., Li J.Q., Wu S.F., Zhu Y.P., Chen Y.W., He F.C. (2006). Integrated nr Database in Protein Annotation System and Its Localization. Comput. Eng..

[104] Finn R.D., Bateman A., Clements J., Coggill P., Eberhardt R.Y., Eddy S.R., Heger A., Hetherington K., Holm L., Mistry J. (2014). Pfam: The protein families database. Nucleic Acids Res..

[105] Pertea M., Pertea G.M., Antonescu C.M., Chang T.C., Mendell J.T., Salzberg S.L. (2015). StringTie enables improved reconstruction of a transcriptome from RNA-seq reads. Nat. Biotechnol..

[106] Trapnell C., Williams B.A., Pertea G., Mortazavi A., Kwan G., van Baren M.J., Salzberg S.L., Wold B.J., Pachter L. (2010). Transcript assembly and quantification by RNA-Seq reveals unannotated transcripts and isoform switching during cell differentiation. Nat. Biotechnol..

[107] Love M.I., Huber W., Anders S. (2014). Moderated estimation of fold change and dispersion for RNA-seq data with DESeq2. Genome Biol..

[108] Benjamini Y., Hochberg Y. (1995). Controlling the False Discovery Rate: A Practical and Powerful Approach to Multiple Testing. J. R. Stat. Soc. Ser. B (Methodol.).

[109] Young M.D., Wakefield M.J., Smyth G.K., Oshlack A. (2010). Gene ontology analysis for RNA-seq: Accounting for selection bias. Genome Biol..

[110] Kanehisa M., Goto S., Kawashima S., Okuno Y., Hattori M. (2004). The KEGG resource for deciphering the genome. Nucleic Acids Res..

[111] Ernst J., Bar-Joseph Z. (2006). STEM: A tool for the analysis of short time series gene expression data. BMC Bioinform..

