# Supplementary material for: Replacing Antibiotics with Synergistic Probiotics–Microalgae Consortium in Mud Crab (Scylla paramamosain) Larviculture: Transcriptomic Evidence for Enhanced Innate Immunity, Oxidative Stress Response, and Metabolic Adaptability
Source: Antibiotics (Basel). 2026 May 16;15(5):498. doi: 10.3390/antibiotics15050498 (PMC13203683; doi:10.3390/antibiotics15050498)
Supplement: Supplementary file 1 [file antibiotics-15-00498-s001.zip › antibiotics-4307061-SI.pdf]

Table S1 Sequencing statistics

Samples		Clean reads	Clean bases	GC Content	%≥Q30	Samples		Clean reads	Clean bases	GC Content	%≥Q30
	AB-1	23,934,685	7,131,347,972	38.53%	95.85%		AB-1	25,323,218	7,525,698,908	42.03%	95.88%
	AB-2	18,309,226	5,460,584,460	38.07%	95.88%		AB-2	25,115,724	7,473,866,422	40.00%	96.12%
	AB-3	20,926,985	6,239,918,874	38.23%	95.13%		AB-3	33,105,991	9,839,041,166	39.23%	96.23%
	CN-1	19,476,474	5,811,380,959	42.24%	95.69%		CN-1	25,448,566	7,580,692,608	42.73%	95.93%
	CN-2	18,436,933	5,493,736,490	38.66%	95.97%		CN-2	26,312,835	7,826,187,236	40.07%	96.09%
	CN-3	17,947,285	5,352,700,908	38.07%	95.82%		CN-3	25,017,414	7,442,092,714	40.01%	96.15%
	MA-1	18,253,764	5,429,546,206	36.98%	95.13%		MA-1	18,903,646	5,617,833,078	39.59%	96.48%
Z1-6h	MA-2	19,803,211	5,904,085,208	36.70%	95.77%	Z3-6h	MA-2	22,043,443	6,567,502,560	40.87%	96.19%
	MA-3	21,283,481	6,344,313,226	37.30%	95.42%		MA-3	19,772,175	5,893,708,392	39.57%	95.77%
	PB-1	21,583,802	6,443,161,668	36.58%	96.25%		PB-1	24,279,578	7,231,996,244	40.06%	96.06%
	PB-2	19,011,680	5,678,650,131	36.32%	95.91%		PB-2	23,382,361	6,966,662,702	39.65%	96.21%
	PB-3	18,086,928	5,387,695,865	35.82%	95.87%		PB-3	19,609,420	5,844,994,278	40.21%	96.00%
	PB-MA-1	18,975,137	5,665,643,562	37.69%	95.71%		PB-MA-1	18,512,918	5,508,059,696	39.42%	96.21%
	PB-MA-2	24,764,277	7,371,872,378	36.49%	95.90%		PB-MA-2	20,454,724	6,083,623,406	40.11%	96.15%
	PB-MA-3	22,788,642	6,793,395,614	36.15%	95.80%		PB-MA-3	19,076,563	5,679,197,430	41.19%	96.06%
	AB-1	21,176,799	6,324,818,042	39.54%	95.86%		AB-1	20,088,594	5,982,699,835	36.38%	96.36%
	AB-2	19,219,424	5,724,393,704	39.75%	95.66%		AB-2	17,783,287	5,304,967,070	36.30%	96.01%
	AB-3	18,022,871	5,379,732,036	37.41%	95.85%		AB-3	22,805,124	6,801,198,506	36.29%	96.34%
	CN-1	19,439,392	5,798,546,836	40.11%	96.01%		CN-1	19,022,981	5,672,963,670	41.19%	96.13%
	CN-2	24,930,186	7,421,301,790	37.72%	95.85%		CN-2	18,820,550	5,591,993,070	41.97%	95.94%
	CN-3	20,330,275	6,061,579,976	39.37%	95.93%		CN-3	19,187,539	5,702,405,990	41.70%	96.11%
	MA-1	19,124,319	5,698,031,974	37.30%	96.18%		MA-1	24,221,390	7,219,729,390	40.53%	96.02%
Z1-24h	MA-2	23,595,588	7,022,988,580	37.04%	96.30%	Z3-24h	MA-2	25,165,615	7,499,268,774	41.58%	95.85%
	MA-3	28,314,011	8,429,567,084	36.76%	96.14%		MA-3	19,740,673	5,881,537,996	41.38%	95.65%
	PB-1	21,845,533	6,514,176,554	38.53%	95.93%		PB-1	21,613,427	6,437,261,040	39.03%	96.01%
	PB-2	22,523,157	6,721,102,156	39.74%	95.66%		PB-2	20,314,718	6,049,704,274	39.14%	95.69%
	PB-3	23,545,321	7,015,603,950	40.78%	95.95%		PB-3	20,240,580	6,029,957,192	40.53%	95.82%
	PB-MA-1	27,823,374	8,306,472,420	37.66%	95.86%		PB-MA-1	19,320,378	5,758,121,250	41.22%	95.76%
	PB-MA-2	26,056,374	7,774,570,920	37.63%	95.96%		PB-MA-2	27,255,334	8,119,043,342	40.61%	96.00%
	PB-MA-3	23,091,165	6,871,986,200	37.01%	96.10%		PB-MA-3	27,520,257	8,190,518,406	40.48%	96.20%

The *Scylla paramamosain* larviculture was affected by different treatments at different developmental stages including Z1 6 hour, Z1 24 hour, Z3 6 hour, and Z3 24 hour. PB, probiotics alone; MA, microalgae alone; PB-MA, probiotics-microalgae consortium; CN, clear water control; AB, antibiotics alone.

Table S2 Function annotation statistics

Annotated Database	Z1-6h		Z1-24h		Z3-6h		Z3-24h	
	Number unigenes	percentage	Number unigenes	percentage	Number unigenes	percentage	Number unigenes	percentage
COG	4829	23.97%	4846	24.00%	4871	24.04%	4852	23.90%
GO	5115	25.39%	5103	25.27%	5132	25.32%	5146	25.35%
KEGG	8764	43.51%	8762	43.39%	8780	43.32%	8804	43.37%
KOG	12248	60.81%	12239	60.61%	12252	60.46%	12260	60.39%
Pfam	13818	68.60%	13832	68.50%	13911	68.64%	13876	68.35%
Swiss-Prot	13457	66.81%	13491	66.81%	13537	66.80%	13537	66.68%
eggNOG	17365	86.21%	17352	85.93%	17437	86.04%	17449	85.96%
NR	19944	99.02%	19990	98.99%	20070	99.03%	20113	99.08%
All_Annotated	20142	100.00%	20194	100.00%	20266	100.00%	20300	100.00%

The *Scylla paramamosain* larviculture was affected by different treatments at different developmental stages including Z1 6 hour, Z1 24 hour, Z3 6 hour, and Z3 24 hour. PB, probiotics alone; MA, microalgae alone; PB-MA, probiotics-microalgae consortium; CN, clear water control; AB, antibiotics alone.

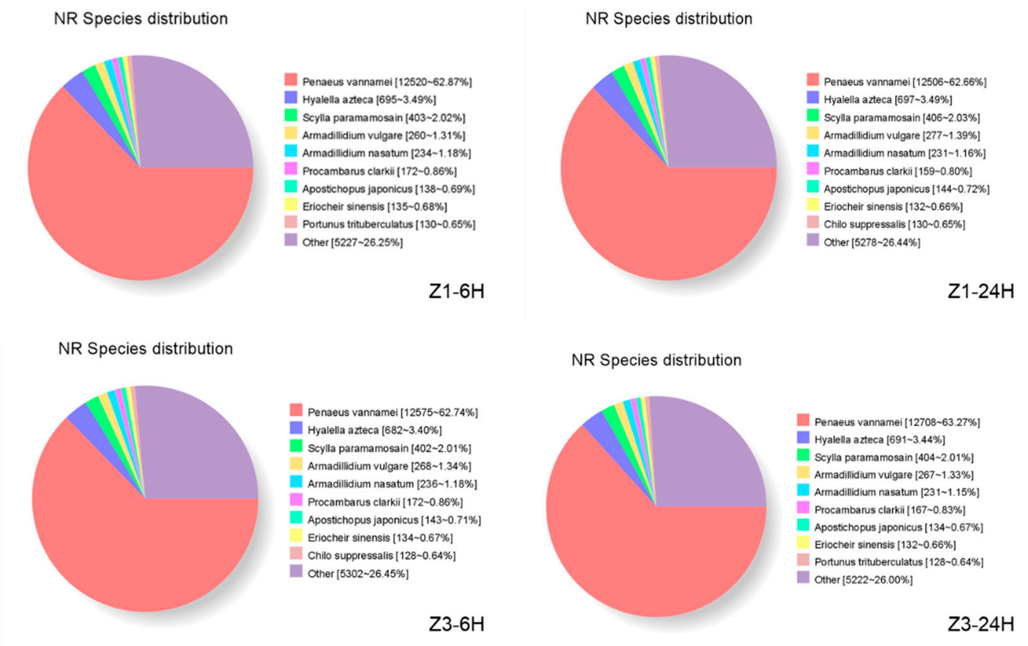

Figure S1 Species distribution in NR annotation. The *Scylla paramamosain* larviculture was affected by different treatments at different developmental stages including Z1 6 hour, Z1 24 hour, Z3 6 hour, and Z3 24 hour.

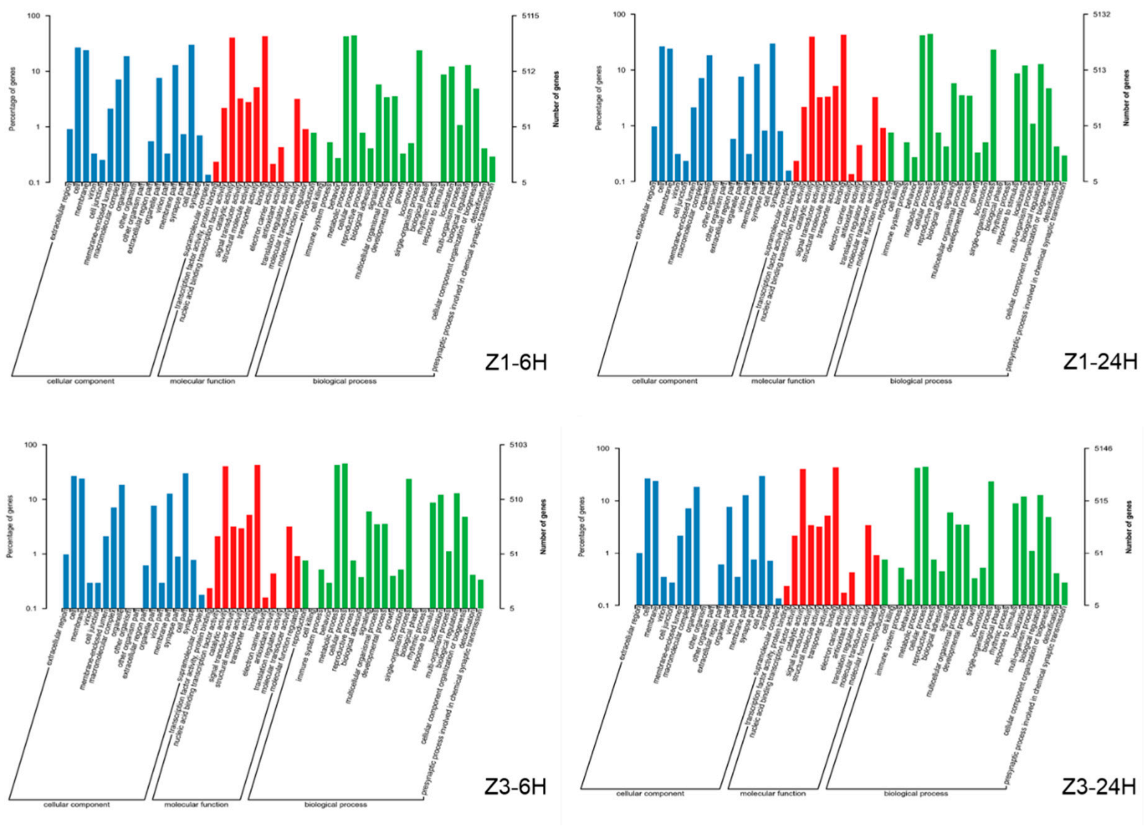

Figure S2 GO classification. The *Scylla paramamosain* larviculture was affected by different treatments at different developmental stages including Z1 6 hour, Z1 24 hour, Z3 6 hour, and Z3 24 hour.

Table S3 Statistics of differentially expressed genes

	DEG Set	DEG Number	up-regulated	down-regulated
Z1-6H	CN_vs_AB	738	99	639
	CN_vs_MA	877	73	804
	CN_vs_PB	878	51	827
	CN_vs_PB-MA	1010	75	935
Z1-24H	CN_vs_AB	374	177	197
	CN_vs_MA	1421	273	1148
	CN_vs_PB	300	140	160
	CN_vs_PB-MA	1308	316	992
Z3-6H	CN_vs_AB	68	32	36
	CN_vs_MA	259	80	179
	CN_vs_PB	62	18	44
	CN_vs_PB-MA	222	69	153
Z3-24H	CN_vs_AB	1907	406	1501
	CN_vs_MA	862	741	121
	CN_vs_PB	826	547	279
	CN_vs_PB-MA	343	245	98

The *Scylla paramamosain* larviculture was affected by different treatments at different developmental stages including Z1 6 hour, Z1 24 hour, Z3 6 hour, and Z3 24 hour. PB, probiotics alone; MA, microalgae alone; PB-MA, probiotics-microalgae consortium; CN, clear water control; AB, antibiotics alone. DGE, differentially expressed gene.
